# Novel, primate-specific PDE10A isoform highlights gene expression complexity in human striatum with implications on the molecular pathology of bipolar disorder

**DOI:** 10.1038/tp.2016.3

**Published:** 2016-02-23

**Authors:** C M MacMullen, K Vick, R Pacifico, M Fallahi-Sichani, R L Davis

**Affiliations:** 1 Department of Neuroscience, The Scripps Research Institute Florida, Jupiter, FL, USA; 2 Department of Informatics Core, The Scripps Research Institute Florida, Jupiter, FL, USA

## Abstract

Bipolar disorder is a highly heritable neuropsychiatric disorder affecting nearly 2.5% of the population. Prior genetic studies identified a panel of common and rare single-nucleotide polymorphisms associated with the disease that map to the first intron of the PDE10A gene. RNA sequencing of striatal brain tissue from bipolar and healthy control subjects identified a novel transcript of PDE10A, named PDE10A19, that codes for a PDE10A isoform with a unique N terminus. Genomic sequences that can encode the novel N terminus were conserved in other primates but not rodents. The RNA transcript was expressed at equal or greater levels in the human striatum compared with the two annotated transcripts, PDE10A1 and PDE10A2. The PDE10A19 transcript was detected in polysomal fractions; western blotting experiments confirmed that the RNA transcript is translated into protein. Immunocytochemistry studies using transfected mouse striatal and cortical neurons demonstrated that the PDE10A19 protein distributes to the cytosol, like PDE10A1, and unlike PDE10A2, which is associated with plasma membranes. Immunoprecipitation and immunocytochemical experiments revealed that the PDE10A19 isoform interacts physically with PDE10A2 and, when expressed at elevated levels, interferes with the plasma membrane localization of PDE10A2. These studies illustrate the complexity of PDE10A gene expression in the human brain and highlight the need to unravel the gene's complex and complete coding capabilities along with its transcriptional and translational regulation to guide the development of therapeutic agents that target the protein for the treatment of neuropsychiatric illness.

## Introduction

Bipolar disorder (BD) is a neuropsychiatric disorder characterized by manic episodes intermixed with periods of depression. Individuals exhibiting mania experience periods of markedly elated moods, are easily distracted and tend to engage in impulsive and high-risk behaviors.^1^ These same individuals then cycle into depressed states of low motivation and lowered hedonia.^2^ Nearly one-third of individuals with BD have attempted suicide.^3^ Family studies show that first-degree relatives of BD subjects have a 5–10% lifetime risk of developing the disease and there is 40–70% concordance among monozygotic twins.^4, 5, 6^ Relatives of BD subjects are also at increased risk for other neuropsychiatric disorders, such as major depression and schizophrenia.^5, 7^ Thus, BD is a highly heritable, chronic neuropsychiatric disorder that affects nearly 2.5% of the population at great expense to the affected individual, family structure and the health-care system.^8^

Genome-wide and case–control association studies have identified numerous susceptibility genes for BD with variants exhibiting modest effect sizes.^5, 9, 10, 11^ We recently queried potential BD-associated loci using a ‘molecular pathway approach,' performing a case–control study focusing on genes that comprise the cAMP pathway,^12^ prompted in part by numerous reports showing disrupted cAMP signaling in BD.^13, 14, 15, 16^ Our results identified a series of common and rare single-nucleotide polymorphisms within the first large intron of the PDE10A gene as associated with BD, which generated great interest given the prominent expression of this enzyme in striatal cells,^17, 18, 19, 20^ the role of the striatum in coordinating and integrating cortical and dopaminergic inputs^21, 22, 23^ and the promise of PDE10A as a pharmacological target for brain disorders including schizophrenia and BD.^24^

PDE10A enzymes contain two GAF (cGMP-stimulated PDEs, *Anabaena* adenylyl cyclases and *Escherichia coli* transcription factor Fhla) domains and hydrolyze both cAMP and cGMP, but have a higher affinity for cAMP.^25^ The PDE10A gene is currently known to be comprised of 23 exons^26^ and maps to chromosome 6q26,^17, 18^ a region previously associated with BD.^1, 12, 27^ The known isoforms of PDE10A, PDE10A1 (refs 17, 18) and PDE10A2,^18, 25^ differ only in their N-terminal sequences owing to alternative splicing and occupy different cellular compartments, with PDE10A2 being primarily membrane-bound and PDE10A1 cytosolic.^28, 29^ The membrane localization of PDE10A2 is due, at least in part, to palmitoylation at Cys-11,^30^ a residue not found at the N-terminal sequences of PDE10A1. Phosphorylation of Thr-16 on PDE10A2 interferes with palmitoylation and redirects the enzyme to the cytosol.^28, 30^

Given the importance of this family of enzymes in neuropsychiatric disorders and the mapping of multiple BD-associated single-nucleotide polymorphisms to the large, first intron of the gene, we pursued experiments to determine how this intronic region might be involved with PDE10A expression. We hypothesized that there may be unknown RNA transcripts with transcriptional start sites and/or coding regions that map to the BD-associated genomic region. We identify here a novel isoform of PDE10A expressed in striatum, named PDE10A19, via RNA sequencing and 5′ RLM-RACE experiments. The PDE10A19 transcript is one of the most abundant PDE10A transcripts in the striatum and it encodes a novel, cytosol-localized isoform with a distinct N terminus. In addition, co-immunoprecipitation experiments reveal that PDE10A19 and PDE10A2 interact physically. Although PDE10A2 localizes to plasma membranes, including those of dendritic spines, the expression level of PDE10A19 influences this localization by redirecting PDE10A2 to the cytosol. These results have broad implications for understanding the subcellular control of cAMP signaling in the striatum, the mechanisms by which PDE10A regulation becomes compromised in brain disorders, and the strategies chosen for developing therapeutics that target PDE10A.

## Materials and methods

An extended description of the Materials and Methods can be found in the Supplementary Information.

Human striatal tissue from four BD subjects and four subjects with no known history of psychiatric disorder (healthy control, HC) were obtained from the Human Brain and Spinal Fluid Resource Center (Los Angeles, CA, USA). The samples were de-identified of all the information other than clinical diagnosis, sex, age at death and postmortem interval (Supplementary Table S1). For RNA sequencing, total RNA was extracted from the putamen and caudate nucleus tissue sections, treated with DNaseI and supplied to The Scripps Research Institute Next Generation Sequencing Core Facility.

The 5′ RNA ligase-mediated rapid amplification of complementary DNA (cDNA) ends (RLM-RACE) studies were performed with HC putamen and a primer that bound within exon 4. The resulting 5′ DNA fragments were cloned and sequenced. To determine whether PDE10A transcripts were alternately spliced between exons 4–22, total RNA was treated with DNAseI and used for cDNA synthesis primed by random hexamers. PCR primer pairs (Supplementary Table S2) were then used to generate overlapping amplicons spanning the downstream PDE10A exons. Randomly primed cDNA was used in real-time PCR assays designed to quantify PDE10A19, PDE10A2 and PDE10A1 transcripts, and the human 18 S rRNA assay was used for normalization.

Polysomes were isolated from four HC striatal tissue specimens, obtained from the Harvard Brain Tissue Resource Center (McLean Hospital, Bellmont, MA, USA), a part of the NIH NeuroBioBank (NIH, Bethesda, MD, USA), via ultracentrifugation on sucrose gradients.^31, 32^ RNA isolation, cDNA synthesis and real-time PCR were performed as described above.

Custom anti-peptide antibodies were generated for each PDE10A isoform by an external contractor. The Scripps Research Institute IACUC approved the production of these antibodies. Full-length PDE10A transcripts with 3′-HA or Flag epitope tags were constructed (Supplementary Table S2) and used to transfect HEK293 for expression studies. Cell lysates were prepared after 2 days using immunoprecipitation buffer with added protease inhibitors by repeated passage through a 26G needle. Western blots were performed using 30 μg of cell lysate fractionated on 4–15% polyacrylamide gels. Western blot membranes were probed with custom PDE10A19, PDE10A2 or PDE10A1 anti-peptide antibodies, anti-Flag polyclonal and an anti-HA monoclonal antibody. Immunoprecipitations (IPs) were performed using 250 μg of cell lysate and 2.5 μg of each specific antibody at 4 °C overnight and then purified using Protein G Plus/Protein A beads.

The PDE10A19-HA or PDE10A2-HA enzyme was affinity purified from transfected HEK293 cell lysates using the HA-tagged Protein Purification Kit (Medical and Biological Laboratories, Aichi, Japan) according to the manufacturer's instructions. Enzymes from four affinity purifications were pooled and desalted by gel filtration to remove excess phosphate from the samples. PDE10A activity was measured within the linear phase of the enzyme's activity over a 15-min time course using the Cyclic Nucleotide Phosphodiesterase Assay Kit (Enzo Life Sciences, Farmingdale, NY, USA) according to the manufacturer's instructions.

Primary neuronal cultures from 0- to 1-day-old C57BL/6J pups were prepared for immunocytochemistry as described.^33^ DIV14–18 primary neurons were fixed, incubated with anti-HA Alexa 488-conjugated monoclonal antibody and/or anti-peptide antibodies (anti-PDE10A1, anti-PDE10A2 and anti-PDE10A19) and imaged.^34^ Immunoreactivity intensity profiles across neurites were computed and normalized for neurite diameter differences and background intensity. The images were analyzed using Image J and statistical analyses performed using Excel and/or Prism.

## Results

### Identification of a novel PDE10A isoform expressed in the human striatum

**Figure 1:**
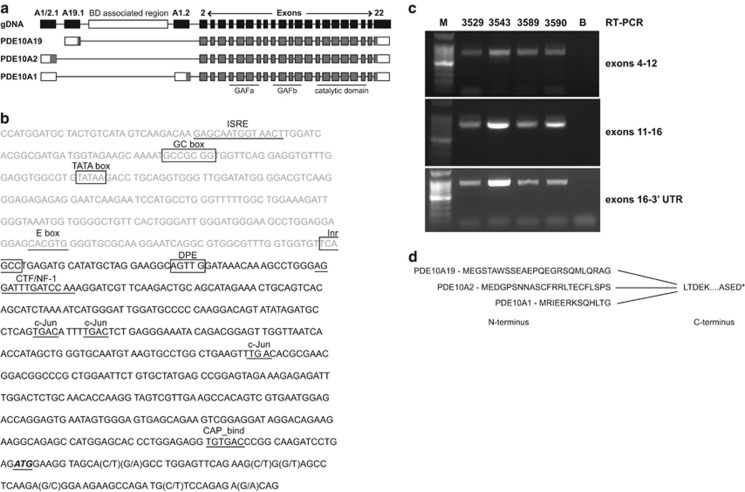
A new PDE10A isoform, PDE10A19, identified from human striatal tissue. (**a**) Gene structure for the PDE10A gene (gDNA). The previously annotated isoforms of PDE10A, PDE10A1 (NR_045597) and PDE10A2 (NM_001130690), are transcribed from the same first exon (A1/2.1). The inclusion of exon A1.2 by alternative splicing distinguishes PDE10A1 from PDE10A2 and introduces an alternative ATG start codon. Open reading frames of the transcripts are shaded gray. The PDE10A19 form (Genbank accession: KU232567) initiates at the A19.1 exon residing between A1/2.1 and A1.2, within the previously defined first intron of the gene. This exon is found relatively close to a genomic region that contains both common and rare variants associated with BD.^12^ Exons 2–22 encode the catalytic domain as well as two GAF domains. PDE10A is transcribed from the minus strand; the 5′→3′ orientation of the sequence is shown. Introns and exons are not drawn to scale. (**b**) PDE10A19 novel exon and promoter sequence. Three hundred bases of promoter sequence are shown in light gray followed by the PDE10A19 novel exonic sequence in black (chr6:166068301-166068824; UCSC genome browser assembly Feb 2009 GRCh37/hg19). A TATA box is located approximately 180 base pairs upstream of the transcriptional start site, labeled Inr (Initiatior); and another core promoter component, the downstream promoter element (DPE), is located 24 bp downstream of the Inr.^37^ Other promoter-associated sequences are boxed and relatively conserved transcription factor binding sites are underlined. The translational start site is bold, italicized and underlined. Within the novel exon coding region, reported SNPs are shown in parenthesis and their genomic location and minor allele frequencies (MAF) are shown in Supplementary Table S5. Bioinformatics analysis was performed with MacVector (MacVector, Cary, NC, USA). (**c**) RT-PCR experiments to generate amplicons from four HC (3529, 3543, 3589, 3590) spanning exons 4–12, 11–16 and 16 3′-UTR. Only one major RT-PCR product was identified for each amplicon on the gel. These amplicons were sequenced, which revealed the anticipated sequence encoded by exons 4 and 22. M, DNA size markers. B, blank control lane. (**d**) The amino acid sequences for the three distinct N termini of PDE10A protein isoforms. All isoforms maintain the same reading frame downstream of the novel N termini. BD, bipolar disorder; RT-PCR, PCR with reverse transcription; SNP, single-nucleotide polymorphism; UTR, untranslated region.

RNA sequencing (RNAseq) was performed on total RNA isolated separately from the putamen and caudate nucleus of postmortem striatal brain tissue from four individuals diagnosed with BD and four individuals with no reported symptoms of any neuropsychiatric disorder (HCs). On average, 20 million reads were obtained from each sample (Supplementary Table S3) and aligned to the human reference genome build GRCh37/hg19. Reads representing the 5′ half of the PDE10A gene (exons 1–4 plus introns; coordinates chr6:166077588-165862416 UCSC genome browser assembly GRCh37/hg19) were apportioned for *de novo* RNA transcript assembly for each of the samples. From this *de novo* alignment, the annotated PDE10A2 transcript (NM_001130690) was detected in all of the HC samples, determined by obtaining reads specific to exon A1/2.1 and spliced to exon 2 (Figure 1a), while the other annotated PDE10A1 transcript (NR_045597) was detected in all but one HC (Supplementary Table S4). Surprisingly, a novel transcript, named PDE10A19, following nomenclature proposed by Beavo, was detected in all of the HC samples (Genbank accession number KU232567, Supplementary Table S4, Figures 1a and b).^35, 36^ This novel first exon resides 6.6 kb downstream of exon A1/2.1 and 111.2 kb upstream of exon 2. We performed 5′ RLM-RACE on HC putamen tissue using a reverse primer within PDE10A exon 4 to confirm the existence of PDE10A19. The 5′ RLM-RACE products were cloned into vectors and directly sequenced. Eleven independent clones, all beginning with the same 5′ nucleotide and containing identical PDE10A19 exon sequence (chr6:166068301-166068824, UCSC genome browser hg19) were found linked to the annotated exons 2, 3 and 4 (Figures 1a and b). A human transcription initiator core promoter element is located at the 5′ nucleotide of the PDE10A19 exon followed by a downstream promoter element consensus sequence (Figure 1b). A TATA box is located 180 base pairs upstream of the initiator core promoter (Inr), which supports this sequence as the authentic transcriptional start site of PDE10A19.

We generated and sequenced overlapping RT-PCR (PCR with reverse transcription) amplicons spanning PDE10A annotated exons 4–22 to ascertain whether any additional RNA transcripts might be produced by alternative splicing across this region. These efforts failed to reveal any major splice variants that arise from the coding region for these exons (Figure 1c). A small fraction of PCR products possessed an alternately spliced PDE10A exon between annotated exons 11 and 12. This exon was found to be either 199 base pairs in size (chr6:165832039-165831841) or 155 base pairs in size (chr6:165831995-165831841) but resulted in PDE10A transcripts predicting a premature stop codon in exon 12 or the novel exon itself, respectively.

Thus, our combined RNAseq and 5′ RLM-RACE data indicate the existence of a novel first exon for PDE10A, a heretofore unknown but predicted protein isoform of the human PDE10A gene, named PDE10A19, and a promoter for the PDE10A gene residing upstream of the novel first exon which may confer different regulatory properties on PDE10A19 expression compared with other PDE10A RNA transcripts. The data further indicate that the N terminus of PDE10A19 is unique but comes into frame with the other PDE10A isoforms at exon 2. Therefore, it is predicted to contain the same GAF binding and catalytic domains found in the PDE10A2 and PDE10A1 isoforms (Figure 1d).

### PDE10A19 is conserved in primates but not rodents

**Figure 2:**
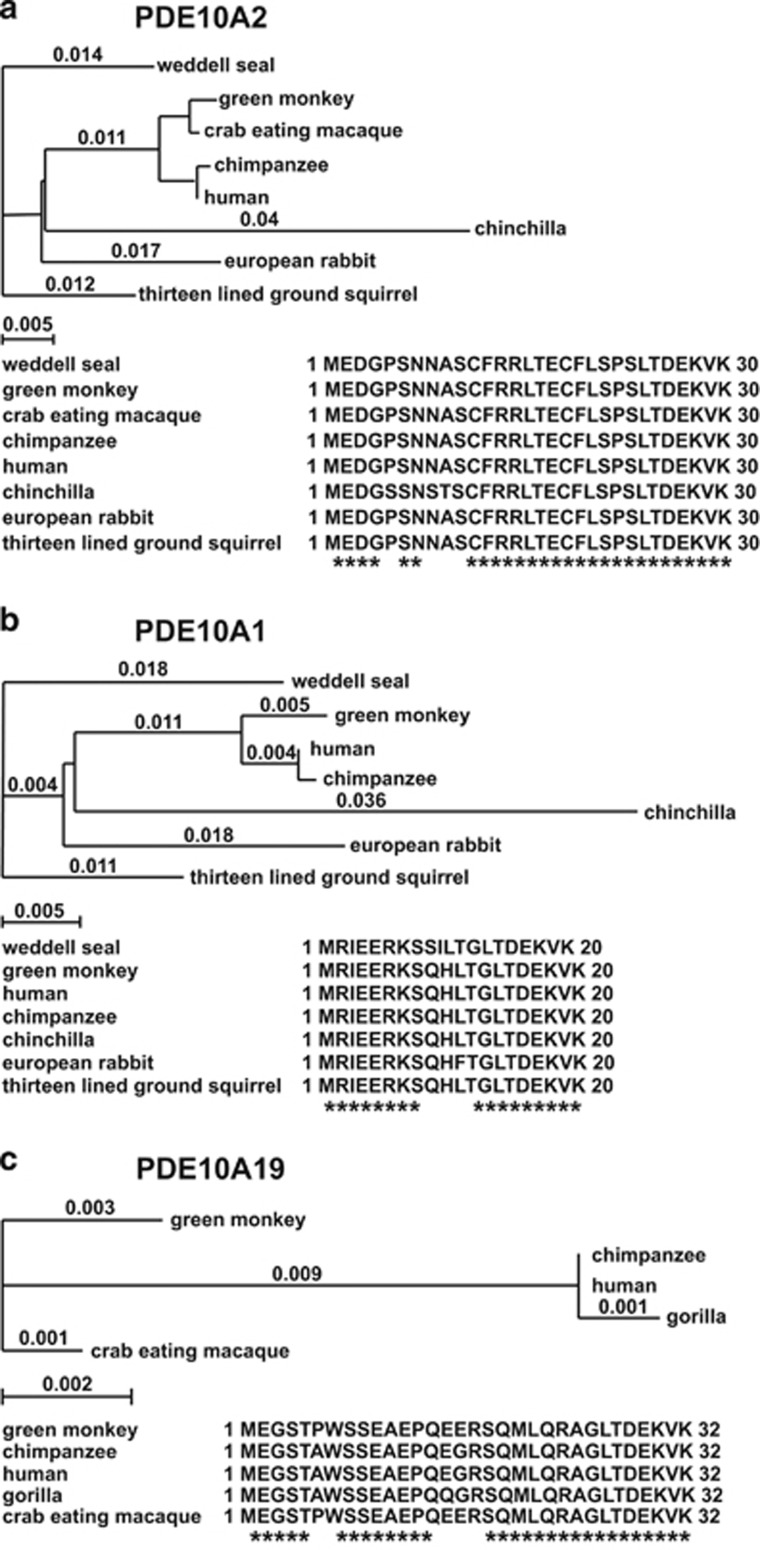
PDE10A isoform homology across species. (**a**) PDE10A2 isoform phylogenic tree analysis of eight organisms showing N-terminal amino acid sequences. The evolutionary distance was calculated using the neighbor-joining; best-tree method with uncorrected *P*-values. Scale bars indicate evolutionary distance between species. (**b**) PDE10A1 isoform phylogenic tree analysis of seven of the eight organisms in **a** along with their N-terminal amino acid sequences. No annotated PDE10A1 isoform sequence was found for the crab-eating macaque. (**c**) PDE10A19 isoform phylogenic tree analysis of five primates along with their N-terminal amino acid sequences.

We queried online protein sequence databases and found that the green monkey and the crab-eating macaque both have predicted proteins that are highly homologous to PDE10A19 (Figure 2). We also translated the first intron of the gorilla and chimpanzee PDE10A genes and discovered genomic sequences that predict the PDE10A19 N-terminal amino acid sequence. This conservation indicates that the PDE10A19 isoform is conserved among these primate species. A similar query of the PDE10A intron sequences from rodent species (rat and mouse) and dog using human PDE10A19 unique sequences failed to find any obvious homology (not shown). Therefore, the PDE10A19 isoform appears to be conserved in the primate order of mammals but not in rodents or other carnivores.

### Expression of PDE10A RNA transcripts

To estimate the abundance of the PDE10A19 transcript relative to PDE10A1 and PDE10A2, we counted the number of RNAseq reads residing within the novel exon of the transcript and normalized these reads for exon size and number of reads that mapped to the upstream PDE10A genomic region used for the *de novo* assembly analysis (Table 1). The PDE10A1 transcript was estimated to be less abundant than both the PDE10A19 (*P*=0.0482) and PDE10A2 (*P*=0.0150) transcripts in the putamen when the data for all the eight subjects were pooled and compared (Supplementary Table S6). The PDE10A19 and PDE10A2 transcripts were not differentially expressed in the putamen. In the caudate nucleus, we found that the PDE10A19 transcript was more abundant than PDE10A2 (*P*=0.0087) and PDE10A1 (*P*=0.0010), while no significant difference was found in the abundance estimate between the PDE10A2 and PDE10A1 transcripts. We also examined the expression level of the three transcripts between the HC and BD subjects. The PDE10A2 transcript was found to be expressed fivefold less abundantly in the BD putamen samples compared with the HC from the RNAseq data (Table 1, *P*=0.0039).

### Expression of PDE10A protein isoforms

We isolated polysomes from postmortem HC striatal tissue to determine whether the three different mRNAs were loaded into protein synthesis machinery. Tissue samples were lysed in the presence of the translational inhibitor cycloheximide and the cytosolic supernatant was separated on a sucrose gradient via ultracentrifugation. The relative transcript abundances in gradient fractions were measured by quantitative RT-PCR. Messenger RNA for all the three PDE10A isoforms, PDE10A2, PDE10A1 and PDE10A19, was detected in both low molecular weight (free RNA) and high molecular weight (containing polysomes) fractions, suggesting their association with polysomes in striatal neurons (Supplementary Figure S1).

**Figure 3:**
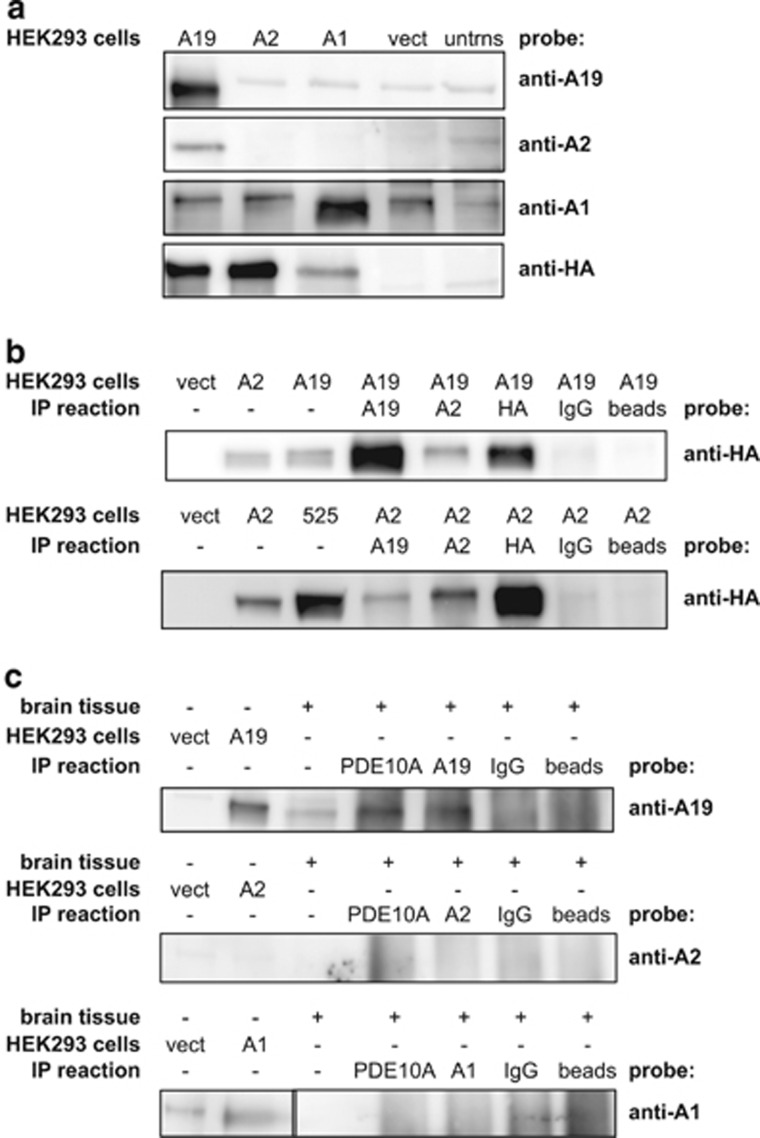
PDE10A19 expression in HEK293 cells and human striatum. (**a**) Western blots of HEK293 cell lysates transfected with constructs for HA-tagged PDE10A19, HA-tagged PDE10A2, HA-tagged PDE10A1 or the empty expression vector. The anti-peptide antibody designed to the novel PDE10A19 isoform binds specifically in HEK293 cells to the HA-tagged PDE10A19 isoform. The anti-peptide antibody designed to the HA-tagged PDE10A2 isoform failed to identify an immunoreactive band in the cells transfected with HA-tagged PDE10A2, but did identify an immunoreactive band in HEK293 cell lysates expressing HA-tagged PDE10A19. The anti-peptide antibody to HA-tagged PDE10A1 identified HA-tagged PDE10A1 as a major immunoreactive band running slightly faster than a nonspecific band observed in all the lanes. The anti-HA antibody identified the three major forms of transfected PDE10A. (**b**) PDE10A19 and PDE10A2 isoform interactions. HEK293 cells were transiently transfected with either the HA-tagged PDE10A19 isoform or the HA-tagged PDE10A2 isoform. Both of these transfected cell lysates were immunoprecipitated (IP) with various antibodies, fractionated by gel electrophoresis and probed with the anti-HA monoclonal antibody. As expected, each N-terminal antibody immunoprecipitated its specific isoform from HEK293 cell lysates and the anti-HA polyclonal also immunoprecipitated each specific isoform. However, the anti-PDE10A2 antibody immunoprecipitated HA-tagged PDE10A19 and the anti-PDE10A19 antibody immunoprecipitated HA-tagged PDE10A2 indicating that these two isoforms can interact. (**c**) PDE10A19 is expressed in human striatum. Human striatal tissue from HC 3589 was used for IP reactions with a commercially available PDE10A antibody (PDE10A-carboxy) that recognizes the carboxy termini of PDE10A isoforms. Isoform-specific, N-terminal anti-peptide antibodies for PDE10A19, PDE10A2 and PDE10A1 were also used for immunoprecipitation and to probe the blots. An immunoreactive protein of the same apparent mass as the HEK293 cell lysate control was detected in immunoprecipitates using the PDE10A-carboxy and PDE10A19 antibodies. No immunoreactivity was observed in any of the lanes using the anti-PDE10A2 anti-peptide antibody. This result was expected on the basis of prior results using transfected HEK293 cell lysates (**a**). No immunoreactivity was observed in any of the lanes except the HEK293 cell control lane using the anti-PDE10A1 anti-peptide antibody, presumably owing to its low abundance of expression. The image for the HEK293 cell lysate lanes was highly contrasted to show the presence of PDE10A1. Images of each full-length western blot on human striatal tissue have been included in Supplementary Figure S10. For HEK293 cells: A1 = cells expressing HA-tagged PDE10A1; vect = cells transfected with the empty vector; A2 = cells expressing HA-tagged PDE10A2; A19 = cells expressing the novel HA-tagged PDE10A19; untrns = untransfected control cells. For IP reactions: A1 = PDE10A1-specific anti-peptide antibody; A2 = PDE10A2-specific anti-peptide antibody; A19 = PDE10A19-specific anti-peptide antibody; IgG = normal rabbit IgG antibody; PDE10A = PDE10A-carboxy; beads = no primary antibody was used for IP, only Protein G Plus/Protein A beads were used for purification. For brain tissue: ‘+' indicates lanes where brain tissue lysates were used; ‘−' indicates where HEK293 cell lysates were added.

We generated anti-peptide antibodies to the unique N termini of the three different PDE10A isoforms to assay their expression in both human tissue and transfected cells. We tested the anti-peptide antibodies using an indirect ELISA assay to determine how the anti-peptide antibodies bound to their specific antigenic peptide (Supplementary Figure S2a). This assay shows that each anti-peptide antibody is specific to its corresponding antigenic peptide compared with other peptides tested. HEK293 cells expressing HA-tagged PDE10A versions of these proteins were also used to test the anti-peptide antibodies. A quantitative RT-PCR assay targeting conserved exons 21 and 22 of the PDE10A gene showed that the HA-tagged transcripts were expressed at levels ranging between 3000 and 5000 times that of empty vector (Supplementary Figure S2c). Western blots were performed with cell lysates expressing each protein isoform (Figure 3) and probed with each individual anti-peptide antibody as well as with a monoclonal antibody against the HA epitope. As anticipated, the anti-HA antibody detected an immunoreactive protein of ~88 kD in cells transfected with the PDE10A isoforms but not in the vector-only or untransfected cells. Similarly, the PDE10A19 and PDE10A1 anti-peptide antibodies identified their respective HA-tagged PDE10A isoforms. Surprisingly, the PDE10A2 anti-peptide antibody failed to highlight the expressed HA-tagged PDE10A2 in cells transfected with HA-tagged PDE10A2, but did highlight a protein in cells transfected with the HA-tagged PDE10A19 construct (Figure 3a). These results cannot be explained by a failure in expression of the HA-tagged PDE10A construct as the quantitative RT-PCR assays (Supplementary Figure S2c) and western blotting experiments using the anti-HA monoclonal antibody demonstrated that the HA-tagged PDE10A2 isoform was expressed at approximately the same levels as the other isoforms (Figure 3a). Rather, these results suggest that the N-terminal epitope of exogenously supplied PDE10A2 is lost or remains unexposed in cells transfected with HA-tagged PDE10A2, and that expression of PDE10A19 stabilizes or exposes the N-terminal epitope from genomically encoded PDE10A2. One explanation for this is that PDE10A2 and PDE1019 form multimers, with the multimer stabilizing or exposing the N-terminal epitope of PDE10A2.

We tested this hypothesis using IP experiments. Cell lysates expressing the HA-tagged PDE10A19 isoform were immunoprecipitated with anti-PDE10A19 anti-peptide antibody, anti-PDE10A2 anti-peptide antibody, anti-HA antibody and normal rabbit IgG. These IP reactions were fractionated and probed with the anti-HA antibody (Figure 3b). This probe highlighted an immunoreactive protein of ~88 kD in all the HEK293 cell positive control lanes but in none of the negative control lanes (Figure 3b), as expected. The PDE10A19 anti-peptide antibody and the anti-HA polyclonal antibody also precipitated the HA-tagged PDE10A19 protein as expected. In addition, the PDE10A2 antibody, likely reacting with genomically encoded PDE10A2, immunoprecipitated the HA-tagged PDE10A19 protein indicating that these two protein isoforms interact physically. The reciprocal IP reaction led to the same conclusion. Immunoprecipitation of genomically encoded PDE10A19 co-precipitated HA-tagged PDE10A2 protein. To verify that the epitope tag did not have a role in PDE10A isoform protein interactions, we also performed immunoprecipitations on protein lysates that were co-transfected HA-tagged PDE10A19 and Flag-tagged PDE10A2 (Supplementary Figure S3a). The anti-Flag IP reaction confirmed that PDE10A19 interacts with PDE10A2 when probed with anti-HA. A reciprocal experiment with Flag-tagged PDE10A19 and HA-tagged PDE10A2 illustrated that anti-Flag immunoprecipitates PDE10A2 (Supplementary Figure S3b); therefore, the proteins interact with each other regardless of which epitope tag is used. Overall, these results indicate that PDE10A2 and PDE10A19 interact physically, either directly or indirectly.

To test whether the PDE10A19 protein is expressed in human brain, we performed IP experiments using human striatal tissue. A commercially available PDE10A antibody that targets the conserved carboxyl terminus of PDE10A was used to IP PDE10A proteins from HC 3589 and the immunoprecipitates were probed with the PDE10A19 anti-peptide antibody. As shown in Figure 3c, the antibody recognizing the C-terminal region of PDE10A proteins immunoprecipitated a protein that is recognized by the PDE10A19 anti-peptide antibody that co-migrated with PDE10A19 expressed in HEK293 cells. In addition, a protein of the same mass was detected directly in striatal tissue by the PDE10A19 anti-peptide antibody. These results provide additional evidence that PDE10A19 is expressed in the human striatum. Parallel IP reactions were performed on HC 3589 striatal tissue using the PDE10A2 and PDE10A1 anti-peptide antibodies. The PDE10A2 anti-peptide antibody failed to detect the PDE10A2 N-terminal epitope in transfected cells, striatal homogenates or the various immunoprecipitates from striatal tissue (Figure 3c). The PDE10A1 anti-peptide antibody detected a protein in transfected HEK293 cells that migrated slightly faster than a nonspecific band that was observed in vector-only transfected cells (Figure 3c, see also Figure 3a), but not in striatal tissue. The PDE10A1 anti-peptide antibody failed to detect the endogenous PDE10A1 in brain tissue, even with IP purification, presumably owing to its low abundance.

To confirm that PDE10A19 hydrolyzed both cAMP and cGMP and exhibited enzymatic properties similar to other PDE10A isoforms, HA-tagged PDE10A19 and HA-tagged PDE10A2 enzymes were affinity purified from transfected HEK293 cell lysates for use in cyclic nucleotide phosphodiesterase assays. Supplementary Figures S4a and c illustrate that both PDE10A19 and PDE10A2 produce increasing amounts of 5′-AMP product across time and that this production is inhibited by the PDE10A-specific inhibitor papaverine. When equal amounts of PDE10A19 enzyme were used to assay both cAMP and cGMP phosphodiesterase activity, PDE10A19 showed more activity towards cAMP as substrate (Supplementary Figure S4b). The calculated IC50 values for papaverine using immunopurified human PDE10A19 and PDE10A2 expressed in HEK293 cells were virtually identical at 73.61 and 76.63 μm, respectively.

We probed the subcellular distribution of the three different forms of PDE10A in transfected cortical and striatal murine neurons by immunocytochemistry and confocal microscopy. Using the anti-HA antibody, we detected the expression of all three forms in striatal neurons (Supplementary Figure S5). The PDE10A1 and PDE10A19 signals were distributed throughout the cytosol and in all neurites, while the PDE10A2 signal was located near cellular membranes. To investigate this difference in localization further, we quantified the signal intensity for all three forms across the diameter of proximal neurites. The PDE10A19 and PDE10A1 signals were distributed as a peak with the maximum intensity near the middle of the neurite profile, consistent with cytosolic localization; whereas the PDE10A2 signal exhibited peaks near the edges of the neurite profile, consistent with membrane-bound localization (Supplementary Figure S5g). Identical results were obtained using transfected cortical neurons (Supplementary Figure S6). We also transfected the HA-tagged PDE10A isoforms in HEK293 cells and isolated proteins in the cytosolic fraction from those in the membrane fraction. Western blots of the fractions for each isoform confirmed the immunocytochemistry findings (Supplementary Figure S7a and b). These results are consistent with prior reports of membrane localization of PDE10A2 and cytosolic localization of PDE10A1.^28, 30^ None of the three isoforms were expressed in the nucleus. (Supplementary Figure S8). We also carefully examined the signal distribution at a subcellular level for all three forms of PDE10A and observed signal for each of the three forms within distal dendritic spines (Supplementary Figure S9). These data suggest a spatially distinct function for the different isoforms, with PDE10A1 and PDE10A19 providing cytosolic PDE activity and PDE10A2 providing membrane-associated PDE activity. All forms may participate in signaling processes within dendritic spines.

**Figure 4:**
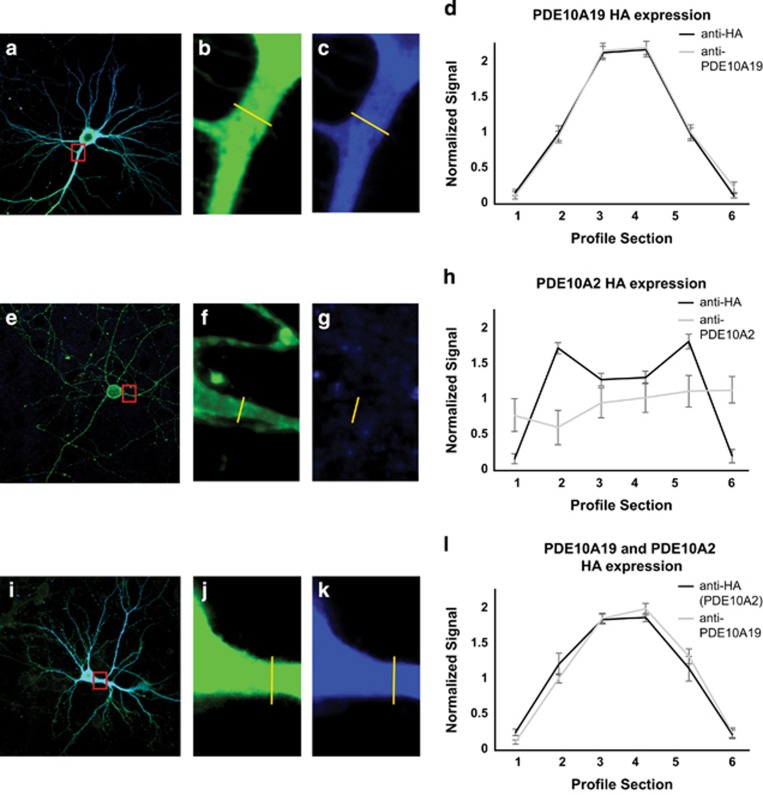
Expression of PDE10A19 alters the subcellular localization of PDE10A2 in mouse cortical neurons. Mouse cortical neurons were transfected with HA-tagged PDE10A19 (**a**–**c**), HA-tagged PDE10A2 (**e**–**g**) and non-HA-tagged PDE10A19 and HA-tagged PDE10A2 (**i**–**k**). Neurons were fixed and stained with either anti-HA antibody conjugated to Alexa 488 and/or with an anti-peptide antibody followed by a goat anti-rabbit Alexa 405 secondary antibody. Red boxes in **a**, **e** and **i** represent the regions enlarged in **b**/**c**, **f**/**g** and **j**/**k**, respectively. Proximal neurites were selected and a line region of interest (ROI) was drawn across the neurites to quantify expression (yellow lines). The ROI intensities of eight neurites for each condition were normalized, binned into six segments, averaged and plotted with error bars representing standard error of the mean (s.e.m.). Plots of the PDE10A19 signal across the neurite exhibit a peak in the center, representing cytosolic localization (**d**). Plots of the PDE10A2 signal exhibit a bimodal distribution with peaks near the cell membrane using an anti-HA primary antibody (**h**). The PDE10A2 anti-peptide antibody failed to detect the PDE10A2 isoform in mouse cortical neurons (**g** and **h**). Plots of co-transfected PDE10A19 and HA-tagged PDE10A2 (**l**). In this situation, the PDE10A19 isoform remains cytosolic, but PDE10A2 also becomes co-localized to the cytosol instead of being directed to the plasma membrane (**l**).

We probed the spatial expression pattern of PDE10A2 when co-expressed with PDE10A19 to determine whether the expression of one isoform influences the spatial localization of the other (Figure 4). In cells transfected with the HA-tagged PDE10A19 isoform, the anti-HA and PDE10A19 anti-peptide antibody signals exhibited a cytosolic localization signature (Figures 4a–d). In cells transfected with the HA-tagged PDE10A2 isoform, the anti-HA signal exhibited apparent membrane localization, whereas little or no signal was detected when the PDE10A2 anti-peptide antibody was used (Figures 4e–h). These results are consistent with previous western blots showing the absence or unexposed nature of the N-terminal epitope of PDE10A2. In cells transfected with equal amounts of the HA-tagged PDE10A2 and a non-HA-tagged PDE10A19 construct, both the PDE10A19 anti-peptide antibody and the anti-HA (representing the PDE10A2 isoform) signals were cytosolic (Figures 4i–l). These results indicate that PDE10A2 becomes localized to the cytosol rather than the membrane when PDE10A19 is expressed at high levels. As the PDE10A19 and PDE10A2 isoforms interact physically, PDE10A19 may interfere with PDE10A2's normal ability to traffic to the plasma membrane, by sequestering it to the cytosol. We confirmed these immunocytochemical results by biochemical fractionation of cytosolic and membrane fractions, and followed the localization of HA-tagged PDE10A2 when co-transfected with PDE10A19. PDE10A2 loses its significant enrichment to the membrane fraction when co-transfected with PDE10A19 (Supplementary Figures S7b and c), confirming the conclusion obtained from the immunocytochemical experiments.

## Discussion

Our prior case–control study of 29 different genes comprising the cAMP signaling pathway identified seven common and eight rare nucleotide variants associated with BD that mapped to a 23 kb intronic region of the PDE10A gene.^12^ Importantly, the rare variants that we identified have not yet been discovered in any of the ongoing genome-sequencing projects, adding weight to the possibility that they are causally associated with BD. This mapping data led to a search for novel exons of PDE10A by sequencing human striatal RNA. Our RNAseq data contained many reads (40–60% of total) that mapped to regions of the genome currently annotated as intronic. Others have reported that up to 64% of the total RNAseq reads map to non-exonic regions of the genome using human brain tissue,^38^ unlike other tissues.^39^ Thus, our data along with others indicate that the complexity of gene expression in the human brain is greatly underappreciated. This dictates a strategy that most, if not all, neurogenetic studies that focus on individual genes should begin with a complete structural characterization of the gene of interest to reduce the possibility of false conclusions.

The strategy applied here to the PDE10A gene revealed unknown complexity in RNA transcripts and the spectrum of protein isoforms encoded by them. The 5′ RLM-RACE experiments using HC putamen tissue confirmed the existence of the PDE10A19 transcript and argued that it includes a unique transcriptional start site and regulatory region, since multiple and independent cDNA clones of PDE10A19 terminated in the identical 5′ nucleotide. The expression of PDE10A19 transcripts was measured at levels equal to or higher than the two annotated forms of PDE10A, PDE10A1 and PDE10A2. Thus, the impact of PDE10A19 on the physiology of human striatal neurons cannot be trivial. This critical fact now needs to underlie all studies of how cAMP signaling influences the physiological state of medium spiny neurons.

A very intriguing observation made was that the genomic sequences encoding the novel N terminus of PDE10A19 is conserved only among primates, and not rodents. This is in contrast to PDE10A1 and PDE10A2, which are conserved isoforms in both primates and rodents. This suggests that the PDE10A19 isoform evolved after the separation of primates from rodents. Species expression differences of PDE isoforms are not unusual; in fact, in the case of the PDE4B4 isoform, while humans and rats have relatively conserved genomic sequences for this isoform, only rats express this protein due to the stop codons positioned in every reading frame of the human nucleotide sequence.^40^ As rodents do not express the PDE10A19 isoform, mouse physiology and behavioral studies become difficult to perform. However, the primate-specific presence of PDE10A19 is consistent with the thought that neuropsychiatric disorders like BD and schizophrenia are human-specific diseases with human-specific etiology.

Besides providing cytosolic PDE activity, PDE10A19 may modulate cAMP signaling by influencing the subcellular localization of its binding partners. PDE10A2 is only one of the three isoforms investigated in this study that appears capable of interacting with the plasma membrane.^25, 28, 30^ Therefore, this isoform may be primarily responsible for submembranous PDE activity, including the PDE10A activity at the membranes of dendritic spines. Our data argue that the three human PDE10A isoforms are non-nuclear, in contrast to some studies of rat non-striatal neurons where PDE10A was primarily localized to the nucleus.^20, 41^ In addition, we show here that PDE10A19 and PDE10A2 interact; and that PDE10A19, when expressed at high levels, can redirect PDE10A2 from a membrane-bound position to a cytosolic one. Thus, the level of expression of PDE10A19 under normal circumstances is predicted to influence the ratio of membrane/cytosolic PDE activity. It also seems possible that mis-expression of PDE10A19 in a diseased state would dysregulate the normal trafficking of PDE10A2, rendering it unable to associate with the plasma membrane and unable to control cAMP signaling at the synapse and/or across cellular membranes. One function of PDE10A activity is to inhibit striatal output by reducing medium spiny neuron excitability.^42^ If this function is primarily because of PDE10A2 membrane localization, then mis-expression of PDE10A19 would be predicted to redirect PDE10A2 to the cytosol, reducing membrane-associated PDE activity, and increasing the excitable state. This idea that a lack of PDE10A enzyme could cause cAMP signaling disruptions has also been suggested as a cause of the motor and psychiatric deficits seen in Huntington's Disease.^43, 44^ Competitive inhibitors of PDE10A have been proposed as therapeutic targets to ameliorate psychosis.^43, 45^ However, if the molecular pathology of psychotic disorders arises from a failure of PDE10A2 to regulate excitability at the membrane, due to mis-expression of PDE10A19, such therapies may be ineffective.

In addition, we discovered that our PDE10A2-specific anti-peptide antibody failed to recognize PDE10A2 protein when expressed alone, but did so when PDE10A19 was expressed. This lack of recognition can be explained by at least two hypotheses: (1) the PDE10A2 anti-peptide antibody fails to recognize the PDE10A2 N terminus because the N-terminal sequences are cleaved from the protein posttranslationally or (2) PDE10A2 palmitoylation at Cys-11 (ref. 30) or phosphorylation at Thr-16 (ref. 28) blocks the anti-peptide antibody epitope. Presumably, the expression of PDE10A19 overcomes the block in antigenic recognition. Mammalian phosphodiesterases exist principally as homodimers, with the exception of PDE1 and PDE6 which are typically heterotetramers.^46, 47^ This multimerization occurs through the GAF-B binding domain of the protein,^48^ and as this region is conserved between the PDE10A19 and PDE10A2 isoforms, it stands to reason that these two isoforms may interact as a heterodimer. The dimerization of PDE10A2 with PDE10A19 may in some way protect or expose the N-terminal antigenic site of PDE10A2.

Our data reveal that the landscape of PDE10A gene expression and protein function in the striatum is much more complex than originally thought. Our studies identify a novel and reasonably abundant form of PDE10A along with its own promoter. We also show that the expression of this isoform influences the subcellular localization of other isoforms, specifically PDE10A2. The cell biological knowledge of this important family of enzymes is critical for understanding the enzyme's role in contributing to neurospsychiatric diseases such as BD. Will the large intronic region found to be associated with BD consist of regulatory elements responsible for controlling the expression of different PDE10A isoforms? As discussed above, disturbances to this balance of transcript/protein abundance could dysregulate cAMP signaling, perhaps in specific subcellular compartments, and alter behavior. Thus, more studies targeted at understanding how these various isoforms are transcriptionally and translationally regulated within the human striatum are warranted.

## Figures and Tables

**Table 1:** PDE10A transcript abundance from RNAseq of human striatal tissue

*Subject*	*Transcript* *5*′*exon size*	*PDE10A19* *525 bp*		*PDE10A2* *150 bp*		*PDE10A1* *278 bp*	
	*PDE10A 5*′*reads*	*Reads*	*Normalized*	*Reads*	*Normalized*	*Reads*	*Normalized*
*Putamen*							
HC 3529	4905	102	3.96E−05	82	1.11E−04	30	2.20E−05
HC 3543	988	33	6.36E−05	15	1.01E−04	1	3.64E−06
HC 3589	3670	123	6.38E−05	34	6.18E−05	2	1.96E−06
HC 3590	6163	153	4.73E−05	53	5.73E−05	45	2.63E−05
HC average	3931.5	102.8	5.36E−05	46	8.29E−05	19.5	1.35E−05
							
BD 3003	4011	96	4.56E−05	10	1.66E−05	6	5.38E−06
BD 4131	1170	8	1.30E−05	4	2.28E−05	4	1.23E−05
BD 4185	8442	152	3.43E−05	32	2.53E−05	29	1.24E−05
BD 4189	2453	51	3.96E−05	2	5.44E−06	4	5.87E−06
BD average	4019	76.8	3.31E−05	12	1.75E−05	10.8	8.98E−06
							
Unpaired *t*-test			*P*=0.0700		*P*=0.0039*		*P*=0.5169

*Caudate*							
HC 3529	2058	19	1.76E−05	5	1.62E−05	3	5.24E−06
HC 3543	9461	144	2.90E−05	38	2.68E−05	13	4.94E−06
HC 3589	490	4	1.55E−05	0	0	0	0
HC 3590	14 841	222	2.85E−05	20	8.98E−06	26	6.30E−06
HC average	6712.5	97.3	2.27E−05	15.8	1.30E−05	10.5	4.12E−06
							
BD 3003	9227	126	2.60E−05	36	2.60E−05	15	5.85E−06
BD 4131	13 230	97	1.40E−05	4	2.02E−06	10	2.72E−06
BD 4185	11 370	188	3.15E−05	16	9.38E−06	1	5.69E−06
BD 4189	1923	29	2.87E−05	1	3.47E−06	1	1.87E−06
BD average	8937.5	110	2.50E−05	14.3	1.02E−05	6.8	4.03E−06
							
Unpaired *t*-test			*P*=0.6600		*P*=0.7376		*P*=1.0000

The reads aligning to the unique exon of each transcript were counted and relative expression levels for the three PDE10A transcripts in the putamen and caudate nucleus were calculated. The ‘PDE10A 5′ reads' column lists the number of reads that aligned to the genomic region (Figure 1) from 1 kb upstream of exon A1/2.1 through exon 4. The ‘HC average' and the ‘BD average' lists the average normalized expression level for the four healthy controls (HCs) and the four bipolar disorder (BD) subjects, respectively. Unpaired, two-sample *t*-tests were performed to determine whether there was a significant difference in expression level between the two groups of subjects; significant differences are indicated with an asterisk.

## Supplementary Material

Supplementary Informations
