# Supplementary material for: Novel, primate-specific PDE10A isoform highlights gene expression complexity in human striatum with implications on the molecular pathology of bipolar disorder
Source: Transl Psychiatry. 2016 Feb 23;6(2):e742–. doi: 10.1038/tp.2016.3 (PMC4872433; doi:10.1038/tp.2016.3)
Supplement: Supplementary Informations [file tp20163x1.docx]

**Materials and Methods**

*Brain tissue*

Human striatal brain tissue sections of four bipolar individuals and four individuals who were not diagnosed with a psychiatric disorder (Healthy Control, HC) were obtained from the Human Brain and Spinal Fluid Resource Center (Los Angeles, CA) and were used in 5’ RLM-RACE and RNA-sequencing studies. All samples were pre-existing and de-identified of all information other than clinical diagnosis, sex, age at death, and postmortem interval (Table S1). An additional four HC samples used for polysome studies were comprised of caudate, nucleus accumbens, and putamen (CAP) tissue obtained from the Harvard Brain Tissue Resource Center (McLean Hospital, Bellmont, MA), a part of the NIH NeuroBioBank (NIH, Bethesda, MD). The Scripps Institutional IRB considered this study exempt human research.

*RNA-sequencing*

Total RNA was extracted from putamen and caudate nucleus tissue sections using the RNeasy lipid tissue mini kit (Qiagen, Venlo, Netherlands). RNA was then treated with DNaseI using the TURBO DNA-free kit (Life Technologies, Carlsbad, CA) and supplied to The Scripps Research Institute Next Generation Sequencing Core Facility (La Jolla, CA). The samples were amplified using the NEBNext Ultra Directional RNA Library Prep Kit for Illumina (New England Biolabs, Ipswich, MA) and were multiplexed with 8 samples run on a single lane of an Illumina HiSeq System (Illumina, San Diego, CA) using 100 bp, single-read chemistry according to manufacturer’s instructions. The RNA-sequencing (RNAseq) reads were aligned to the human reference genome and transcriptome (hg19) by the Avadis NGS 1.5.1 software (Strand Scientific Intelligence, Inc., San Francisco, CA), using the Ensembl gene and transcript annotation (2013.04.03) option. The minimum percent identity was set to 90 and reads with quality scores less than 30 were trimmed at the 3’ end. *De novo* alignment of reads mapping to coordinates chr6:166077588-165862416 (UCSC genome browser assembly Feb 2009 GRCh37/hg19) was performed on each sample using Sequencher v5.2.3 (Gene Codes Corporation, Ann Arbor MI). RNAseq transcript abundance was calculated by normalizing the number of reads mapping to the unique 5’ exon of the transcript and dividing this value by the 5’ exon size (base pairs) multiplied by the number of reads that were aligned to the overall PDE10A coordinate region used for the *de novo* alignment. An unpaired t-test was used to determine whether transcript abundance was significantly different between HC and BD groups for each transcript. A repeated measures ANOVA test, followed by a Tukey’s post hoc test, was performed to determine whether the PDE10A transcripts were differentially expressed. Raw RNAseq data will be made available upon request.

*Reverse transcriptase PCR (RT-PCR)*

The 5’ RNA ligase mediated rapid amplification of cDNA ends (RLM-RACE) studies were performed on HC putamen tissue using the FirstChoice RLM-RACE kit (Life Technologies) according to manufacturer’s instructions. This modified version of the classic RACE protocol selects for mature RNA species [^1^](#_ENREF_1)^,^ [^2^](#_ENREF_2). The resulting 5’ DNA fragments were cloned using the TA cloning kit (Life Technologies) according to manufacturer’s instructions and sequenced. To determine if the PDE10A transcript had alternately spliced isoforms involving exons 4-22, total RNA (250 ng) from both putamen and caudate nucleus tissue was treated with DNAseI and used for cDNA synthesis primed by random hexamers using the SuperScript III first-strand synthesis system (Life Technologies). PCR primer pairs were used to generate overlapping amplicons spanning the downstream PDE10A exons (primer sequences are presented in Table S2). The PCR products were then verified by DNA sequencing.

*Real time PCR (qRT-PCR)*

Randomly primed cDNA was generated, as described above, for use in real time PCR assays (Life Technologies) specifically designed to quantitate the PDE10A19, PDE10A2, and PDE10A1 transcripts according to manufacturer’s instructions. The human 18S rRNA assay was used to calculate normalized delta Ct values for these analyses.

*Polysome analyses*

Five hundred mg of striatal tissue from four different HC samples were used for polysome preparations. Freshly dissected mouse brain tissue was prepared in parallel as a control. All materials and buffers were kept on ice unless otherwise specified. Samples were placed in HHBSS buffer (1x Hank’s basal salt solution, 2.5 mM HEPES-KOH pH 7.4, 35 mM glucose, 4 mM NaHCO_3_) containing 100 μg/mL of cycloheximide (CHX; Sigma, St. Louis, MO). Each sample was dissected free of white matter and transferred to a tube with 600 mL of homogenization buffer (10 mM HEPES-KOH pH 7.4, 150 mM KCl, 5 mM MgCl_2_, 100 μg/mL CHX and 0.5 mM dithiothreitol) containing 1X protease inhibitor cocktail (ThermoScientific, Waltham, MA), 1% v/v phosphatase inhibitor cocktails 2 and 3 (Sigma) and 100 U/mL RNAse inhibitor (SUPERase-In, Life Technologies). Tissues were manually homogenized and incubated on ice for 5 min in buffer containing 1% v/v NP-40 detergent. The samples were then centrifuged at 20,000 x g for 10 min at 4°C. The supernatant for each sample was supplemented with 40 U of RNAse inhibitor and divided into two aliquots. One aliquot received EDTA to a final concentration of 50mM, while the other received the same volume of water. Samples were incubated for 1 h at room temperature and loaded onto 11 mL 15%-50% w/w sucrose gradients, that had been previously made in homogenization buffer, stored at -80°C, and thawed at 4°C for 3 h prior to centrifugation. The loaded gradients were centrifuged at 40,000 rpm for 2 h at 4°C in a SW 41 rotor (Beckman Instruments, Indianapolis, IN). Fractions of 625 μL were collected by volume displacement with fluorinert (FC-40, Sigma) and an automated syringe pump with continuous monitoring by UV absorption at 254 nm. Fractions 1, 2 and 3 were combined as a low molecular weight (LMW) pool. Fractions 9, 10 and 11 were combined as a high molecular weight (HMW) pool. RNA isolation, cDNA synthesis, and real time PCR were performed as described above. The GAPDH and 18S rRNA assays (Life Technologies) were used as endogenous controls. The relative abundance for each transcript was calculated by normalizing their average Ct values against PDE10A2. The ratio of transcript levels between the free RNA and polysome pools was determined by subtracting the Ct values of the former from the latter. The effect of the ribosome-destabilizing EDTA treatment on transcript distribution was measured by a change in this second ΔCt.

*Antibodies*

Custom anti-peptide antibodies were generated for each PDE10A isoform by New England Peptide (Gardner, MA). The Scripps Research Institute IACUC approved the production of these antibodies. Peptides ranging from between 13-17 amino acids were chosen from the sequence predicted from the first unique exon of each transcript. These peptides were conjugated to keyhole limpet hemocyanin (KLH) and injected into New Zealand White rabbits three times over a period of 28 days. After day 35, production bleeds were affinity purified and the resulting antibodies were used for western blotting, immunoprecipitation, and immunocytochemistry experiments. The custom anti-peptide antibodies were tested for specificity via an indirect ELISA protocol. Twenty micrograms of each antigenic peptide and two non-specific peptides to *D. melanogaster* proteins were coated overnight on 96 well plates using 100mM bicarbonate/carbonate coating buffer. Plates were washed three times with 200 μL 1X TBST buffer and blocked with a 5% non-fat dry milk/1X TBST solution for two hours at room temperature. Plates were washed three times with 200 μL 1X TBST buffer and incubated with a 100 μL 1:1000 dilution of each anti-peptide primary antibody or normal rabbit IgG antibody (Cell Signaling Technology, Danvers, MA) for two hours at room temperature. Plates were washed four times with 200 μL 1X TBST buffer and 100 μL of a 1:5000 dilution of anti-rabbit horse-radish peroxidase conjugated secondary antibody (Clontech, Mountain View, CA) was incubated at room temperature for two hours. Plates were washed five times with 200 μL 1X TBST buffer and 100 μL of 1-Step Turbo TMB-ELISA substrate (ThermoScientific) was added. After 5 min, 100 μL of 2M H_2_SO_4_ stop solution was added and the absorbance at 450 nm was recorded.

*Transfections*

A pCDNA3.1-based expression vector (pCAGWBA) containing a CAG promoter and WPRE was designed for expressing PDE10A isoforms in mammalian culture cells. Each specific transcript from its translational start to its stop codon was isolated from HC putamen cDNA via PCR and cloned into the vector (primer sequences are presented in Table S2). We also inserted an HA or a Flag epitope tag just upstream of the stop codon (see Figure S2b for vector map). The HA/Flag-tagged PDE10A constructs were used to transfect HEK293 cells (ATCC, Manassas, VA) using the Polyfect transfection reagent (Qiagen) according to manufacturer’s instructions. After 48 h, the cells were harvested and cell lysates were prepared for immunoprecipitation studies using immunoprecipitation buffer (50 mM Tris pH 7.4, 150 mM NaCl, 1 mM EDTA, 1% Chaps, 0.1 mg/mL BSA) with 1X protease inhibitor cocktail (added fresh) by repeated passage through a 26G needle. For cellular localization studies, cytosolic and membrane cell lysates were prepared using the Mem-PER Plus membrane protein extraction kit (ThermoScientific) according to manufacturer’s instructions.

*Western blotting and immunoprecipitation*

Western blots were performed using 30 µg of cell lysate fractionated on 4-15% polyacrylamide gels (Bio-Rad Laboratories, Hercules, CA) for 2 h at 150V. Proteins were transferred to PVDF membranes using the Transblot SD semi-dry transfer cell (Bio-Rad Laboratories) at 11 V for 40 min. Blots were incubated with a 1:1000 dilution of primary antibody in antibody binding buffer (1X TBST buffer with 4% fish gelatin, Sigma, St. Louis, MO) at 4°C overnight. Primary antibodies included the custom anti-PDE10A19, anti-PDE10A2, and anti-PDE10A1 anti-peptide antibodies (New England Peptide), an anti-Flag polyclonal antibody (ThermoScientific) and an anti-HA monoclonal antibody (Covance, Princeton, NJ). Blots were washed five times with 1X TBST buffer and incubated with a 1:5000 dilution of a horseradish peroxidase conjugated secondary antibody (Clontech) in the antibody binding buffer at room temperature for 1 h. Blots were washed five times and visualized with a chemilluminescent imager (Alpha Innotech, San Leandro, CA) after development with an ECL substrate (Advansta, Menlo Park, CA). Image intensity values were determined using ImageJ (NIH, Bethesda, MD) and statistics calculated with Prism (GraphPad, San Diego, CA). Immunoprecipitations (IP) were performed on 250 μg of cell lysate that was pre-incubated with 50 μL of Protein G Plus/Protein A beads (EMD Millipore, Billerica, MA) to reduce the non-specific binding in the reaction. The cell lysates were then immunoprecipitated using 2.5 μg of each specific antibody at 4°C overnight. Antibodies used for immunoprecipitation included the custom anti-PDE10A19, anti-PDE10A2, and anti-PDE10A1 anti-peptide antibodies (New England Peptide); an anti-Flag polyclonal antibody (ThermoScientific); an anti-HA polyclonal antibody (Clontech); a commercial anti-PDE10A antibody (Scottish Biomedical, Glasgow, Scotland, United Kingdom) and normal rabbit IgG (Cell Signaling Technology). IP reactions were purified using the Protein G Plus/Protein A beads according to manufacturer’s instructions and fractionated on Western blots as described above.

*Enzyme assays*

Confluent plates of HEK293 cells were transfected with either pCAGWBA-PDE10A19-HA or pCAGWBA-PDE10A2-HA as described above. For PDE10A19 assays, two 10mm^2^ plates of confluent cells were harvested and lysed in immunoprecipitation buffer in a total volume of 500 µL; while for PDE10A2 assays, three 10 mm^2^ plates of confluent cells were harvested and lysed in a total volume of 500 μL. PDE10A19-HA or PDE10A2-HA enzyme was affinity purified from these 500 μL volume lysates using the HA-tagged Protein Purification Kit (Medical & Biological Laboratories, CO., LTD, Aichi, Japan) according to manufacturer’s instructions. Enzyme from four affinity purifications were pooled and desalted in a P6 DG gel filtration column (Enzo Life Sciences, Farmingdale, NY) to remove excess phosphate from the samples. PDE10A activity was measured using 200 μM cyclic nucleotide over a 15 min time course using the Cyclic Nucleotide Phosphodiesterase Assay Kit (Enzo Life Sciences) according to manufacturer’s instructions. Enzyme assays were kept within the linear phase for cNMP hydrolysis (<15% substrate utilized). The following final concentrations of papaverine hydrochloride (Sigma) were used to determine the IC50 for PDE10A19 and PDE10A2: 300 μM, 100 μM, 20 μM, and 1.7 μM. After adding enzyme and allowing the reaction to proceed for 10 min, the 5’-NMP produced was measured and used to generate a plot of % inhibition versus papaverine concentration. A best-fit line was generated from the plot and the function used to calculate the IC50 for both PDE10A19 and PDE10A2.

*Immunocytochemistry*

Primary neuronal cultures from 0-1 day-old C57BL/6J pups were prepared by rapidly dissecting forebrain tissue in ice-cold Hank’s balanced salt solution (HBSS, Life Technologies). Brain tissue was partially digested by papain (Worthington, Lakewood Township, NJ), washed, triturated, and plated on a poly-d-lysine (Sigma) coated 384 well plates (Greiner, Monroe, NC) at a density of 12,000 cells per well. Plating media contained 5% fetal bovine serum, 2% Glutamax and 0.02% gentamycin in NeuroBasal (all from Life Technologies). Four h after plating, media was replaced with serum-free NeuroBasal-A media containing 1% Glutamax, 2% B27 and 0.02% gentamycin. A 50% media change was performed every 4-5 days and 0.2% 10uM 5-Fluoro-2′-deoxyuridine (Sigma) was added at DIV4 to prevent glial cell overgrowth. Transfections were performed at DIV4-6 using Lipofectamine 2000 (Life Technologies). DIV14-18 primary neurons were fixed at room temperature with 4% paraformaldehyde (Electron Microscopy Sciences, Hatfield, PA) for 20 min and washed 3 times with 0.3% Triton-X PBS (TPBS) and 0.3 M glycine (Sigma). A 30 min block with 0.3% Triton-X, 0.5% BSA (Sigma) and 5% Goat Serum (Sigma) was followed with an overnight incubation at 4°C with primary antibodies. Wells were washed 6 times with TPBS before the secondary antibodies were applied in blocking buffer. Primary antibodies were anti-HA Alexa 488 conjugated monoclonal antibody (Life Technologies) at a 1:100 dilution; and anti-PDE10A1, anti-PDE10A2, and anti-PDE10A19 anti-peptide antibodies all at a 1:3000 dilution. Goat anti-rabbit Alexa 405 (Life Technologies) was used as a secondary antibody at a 1:400 dilution. An InCell 6000 confocal screening microscope (GE Healthcare, Boston, MA) was used to collect images. Immunoreactivity intensity profiles of neurite cross sections were computed by drawing a line with a width of a single-pixel across the neurite. These cross-sectional regions of interest included a small line segment on either side of the neurite imaged. The plots representing cross-section intensity were partitioned into six equal sections to normalize for neurite diameter differences and the average pixel intensity per section calculated. The background intensity was subtracted and the average pixel intensity of each section divided by the average pixel intensity across the entire region of interest to normalize for differences in intensity levels between cells. The normalized intensity levels by section were averaged across 8 different cell neurites and presented as the cross-sectional intensity plots in the figures. Error bars represent the standard error of the mean. Images were analyzed using Image J (NIH) and statistical analyses performed using Excel (Microsoft, Redmond, WA) and/or Prism (GraphPad).

**Materials and Methods References**

1. Maruyama K, Sugano S. Oligo-capping: a simple method to replace the cap structure of eukaryotic mRNAs with oligoribonucleotides. *Genes* 1994; **138:** 171-174.

2. Shaefer B. Revolution in rapid amplification of cDNA ends: new strategies for polymerase chain reaction cloning of full-length cDNA ends. *Analytical Biochem* 1995; **227:** 255-273.
