# Supplementary material for: Novel, primate-specific PDE10A isoform highlights gene expression complexity in human striatum with implications on the molecular pathology of bipolar disorder
Source: Transl Psychiatry. 2016 Feb 23;6(2):e742–. doi: 10.1038/tp.2016.3 (PMC4872433; doi:10.1038/tp.2016.3)
Supplement: Supplementary Table S1 [file tp20163x2.docx]

| **ID** | **Clinical Diagnosis** | **Sex** | **Age at Death** | **Post Mortem Interval** |
| --- | --- | --- | --- | --- |
| **3529** | HC | M | 58 | 9.0 |
| **3543** | HC | F | 73 | 12.0 |
| **3589** | HC | M | 53 | 15.0 |
| **3590** | HC | M | 75 | 11.5 |
| **3003** | BD | F | 44 | unknown |
| **4131** | BD | F | 57 | 15.8 |
| **4185** | BD | M | 73 | 11.7 |
| **4189** | BD | F | 45 | 18.3 |

**Table S1. Clinical features of post-mortem brain tissue.** This table illustrates the clinical diagnosis, sex, age at death, and post mortem interval (h) before the brain tissue was harvested from each individual.
