# Supplementary material for: Novel, primate-specific PDE10A isoform highlights gene expression complexity in human striatum with implications on the molecular pathology of bipolar disorder
Source: Transl Psychiatry. 2016 Feb 23;6(2):e742–. doi: 10.1038/tp.2016.3 (PMC4872433; doi:10.1038/tp.2016.3)
Supplement: Supplementary Table S2 [file tp20163x3.docx]

| **Primer name** | **Primer sequence (5’ 🡪 3’)** |
| --- | --- |
|  | |
| **Primers used to assay PDE10A alternative splicing at the 3’ end of the transcript** | |
| PDE10A exon 4F | GCTACTCCTCTATGAACTGAGCAG |
| PDE10A exon 12R | GACTTCCCCTGTTCTTGCTACT |
| PDE10A exon 11F | AAAACCTGGTGAATGCCG |
| PDE10A exon 16R | CAAGGTCTGTGAAAAGCGTG |
| PDE10A exon 16F | TCTGTGAAGAAGAACTATCGGC |
| PDE10A 3'UTR R | ATGTCAAAGAAGCAAGATGAGG |
|  |  |
| **Primers used for PDE10A transcript cloning** | |
| PDE10A19 FseI FOR | CCGGCAAG**GGCCGGCC**ATGGAAGGTAG |
| PDE10A2 FseI FOR | TGCTCTTC**GGCCGGCC**GACATGGAAGATGGACC |
| PDE10A1 FseI FOR | AGGAAAA**GGCCGGCC**AATGAGGA |
| PDE10A HA FseI REV | GTCAGGGT**GGCCGGCC**CTCA*AGCGTAATCTGGAACATCGTATGGGTA*ATCTTCAGATGCAGC |
| PDE10A Flag FseI REV | ATCGAATTCCTGCAG**GGCCGGCC**CTCA*CTTATCGTCGTCATCCTTGTAATC*ATCTTCAGATGCAGC |

**Table S2. PCR primers used to survey for PDE10A alternative splicing and transcript cloning**. The primers used to assay whether PDE10A transcripts are alternatively spliced in their 3’ half are shown. The primers used to clone full-length PDE10A transcripts into a mammalian expression vector used for transfection studies are also shown. The FseI restriction enzyme site is bolded and the ATG start or TGA stop codons are underlined. The HA or Flag epitope tag is italicized.
