# Supplementary material for: Novel, primate-specific PDE10A isoform highlights gene expression complexity in human striatum with implications on the molecular pathology of bipolar disorder
Source: Transl Psychiatry. 2016 Feb 23;6(2):e742–. doi: 10.1038/tp.2016.3 (PMC4872433; doi:10.1038/tp.2016.3)
Supplement: Supplementary Table S3 [file tp20163x4.docx]

| **Putamen** |  | **HC 3529** | **HC 3543** | **HC 3589** | **HC 3590** |
| --- | --- | --- | --- | --- | --- |
|  | Total number of reads | 24 103 338 | 5 495 047 | 13 256 121 | 19 210 605 |
|  | Aligned reads | 16 615 234 | 4 649 139 | 10 109 911 | 13 078 981 |
|  | Aligned to transcriptome only | 6 910 340 | 2 493 335 | 3 900 997 | 4 839 579 |
|  | Aligned to introns | 9 703 817 | 2 155 332 | 6 208 300 | 8 238 701 |
|  | Unaligned reads | 7 488 104 | 845 908 | 3 146 210 | 6 131 624 |
| **Caudate** | Total number of reads | 19 401 146 | 19 652 608 | 19 626 286 | 18 602 822 |
|  | Aligned reads | 16 369 882 | 16 754 514 | 15 581 626 | 16 239 952 |
|  | Aligned to transcriptome only | 6 500 485 | 6 555 024 | 4 775 291 | 5 344 103 |
|  | Aligned to introns | 9 868 649 | 10 198 678 | 10 805 880 | 10 895 110 |
|  | Unaligned reads | 1 922 718 | 1 876 316 | 2 859 447 | 1 378 878 |
|  |  |  |  |  |  |
| **Putamen** |  | **BD 3003** | **BD 4131** | **BD 4185** | **BD 4189** |
|  | Total number of reads | 5 165 166 | 30 016 071 | 41 526 635 | 23 406 928 |
|  | Aligned reads | 4 203 978 | 20 279 985 | 31 136 952 | 16 560 378 |
|  | Aligned to transcriptome only | 1 598 443 | 3 722 703 | 13 550 144 | 2 835 493 |
|  | Aligned to introns | 2 605 314 | 16 556 911 | 17 584 110 | 13 724 618 |
|  | Unaligned reads | 961 188 | 9 736 086 | 10 389 683 | 6 846 550 |
| **Caudate** | Total number of reads | 18 515 367 | 20 741 185 | 21 742 149 | 23 372 769 |
|  | Aligned reads | 15 902 076 | 17 497 438 | 18 500 754 | 16 769 422 |
|  | Aligned to transcriptome only | 6 219 339 | 5 337 821 | 7 014 531 | 2 469 950 |
|  | Aligned to introns | 9 682 001 | 12 159 247 | 11 485 074 | 14 299 202 |
|  | Unaligned reads | 1 626 838 | 2 084 298 | 2 082 113 | 5 124 911 |

**Table S3. Statistics for RNAseq data of human striatal tissue**. The eight total RNA samples from putamen were multiplexed so that each sample would generate approximately 20 million reads. The eight caudate nucleus samples were prepared similarly. Resulting raw data was aligned to the human reference genome and transcriptome (hg19).
