# Supplementary material for: Novel, primate-specific PDE10A isoform highlights gene expression complexity in human striatum with implications on the molecular pathology of bipolar disorder
Source: Transl Psychiatry. 2016 Feb 23;6(2):e742–. doi: 10.1038/tp.2016.3 (PMC4872433; doi:10.1038/tp.2016.3)
Supplement: Supplementary Table S4 [file tp20163x5.docx]

|  | **Exons included** | **HC 3529** | **HC 3543** | **HC 3589** | **HC 3590** | **BD 3003** | **BD 4131** | **BD 4185** | **BD 4189** |
| --- | --- | --- | --- | --- | --- | --- | --- | --- | --- |
| **Putamen** |  |  |  |  |  |  |  |  |  |
| PDE10A19 | A19.1🡪2,3,4 | + | + | + | + | + | - | + | + |
| PDE10A2 | A2.1🡪2,3,4 | + | + | + | + | + | - | + | - |
| PDE10A1 | A1.1🡪2,3,4 | + | - | - | - | + | - | - | - |
| **Caudate** |  |  |  |  |  |  |  |  |  |
| PDE10A19 | A19.1🡪2,3,4 | + | + | - | + | + | + | + | + |
| PDE10A2 | A2.1🡪2,3,4 | + | + | - | + | + | + | - | - |
| PDE10A1 | A1.1🡪2,3,4 | - | + | - | + | - | + | + | - |

**Table S4. PDE10A transcripts found via RNAseq *de novo* alignment**. Reads mapping to chr6:166077588-165862416 of UCSC genome browser assembly GRCh37/hg19 were aligned against one another and resulting consensus sequences were analyzed for the presence of PDE10A spliced transcripts. The total number of reads used for *de novo* analysis can be found on Table 1. Not only were the annotated PDE10A2 and PDE10A1 transcripts detected, but a novel transcript, PDE10A19, was detected. The presence (indicated by a “+”) or absence (indicated by a “-“) of each transcript is indicated for each individual analyzed.
