# Supplementary material for: Novel, primate-specific PDE10A isoform highlights gene expression complexity in human striatum with implications on the molecular pathology of bipolar disorder
Source: Transl Psychiatry. 2016 Feb 23;6(2):e742–. doi: 10.1038/tp.2016.3 (PMC4872433; doi:10.1038/tp.2016.3)
Supplement: Supplementary Table S5 [file tp20163x6.docx]

| **SNP** | **gDNA**  **position** | **cDNA**  **position** | **cDNA**  **substitution** | **a.a.**  **position** | **a.a.**  **substitution** | **MAF** | **SIFT** |
| --- | --- | --- | --- | --- | --- | --- | --- |
| rs114518404 | 166068359 | 14 | G/T | 5 | Thr-Met | 0.90 | NT |
| rs142993564 | 166068358 | 15 | G/A | 5 | Thr-Thr | 0.16 | ND |
| rs148579376 | 166068341 | 32 | G/T | 11 | Ala-Val | 0.36 | T |
| rs880122 | 166068340 | 33 | G/T | 11 | Ala-Ala | 9.66 | ND |
| rs880121 | 166068328 | 45 | G/C | 15 | Glu-Asp | 33.23 | NT |
| rs568000059 | 166068313 | 61 | C/T | 21 | Leu-Phe | 0.02 | T |
| rs188852383 | 166068303 | 70 | G/A | 24 | Ala-Thr | 0.02 | NT |

**Table S5. PDE10A19 coding region SNPs**. A total of 57 SNPs have been reported for the genomic sequence shown in Figure 1b (dbSNP 142). Forty-eight of these SNPs had allele frequency data showing that 41 of them would be considered rare variants (with minor allele frequencies less than 1%). The seven SNPs shown above are located in the coding region of the PDE10A19 novel exon. Listed are their genomic (gDNA) location, their cDNA position and substitution, their amino acid (a.a.) position and substitution as well as their minor allele frequency (MAF) in percent. Of the 7 SNPs listed, 5 of them are rare variants. For any missense substitutions, their probability of affecting the protein’s function was surveyed using SIFT (http://sift.bii.a-star.edu.sg/) where NT = not tolerated, ND = not determined, and T = tolerated.
