# Supplementary material for: Novel, primate-specific PDE10A isoform highlights gene expression complexity in human striatum with implications on the molecular pathology of bipolar disorder
Source: Transl Psychiatry. 2016 Feb 23;6(2):e742–. doi: 10.1038/tp.2016.3 (PMC4872433; doi:10.1038/tp.2016.3)
Supplement: Supplementary Table S6 [file tp20163x7.docx]

| **Tissue** | **PDE10A19**  **normalized expression average** | **PDE10A2**  **normalized expression average** | **PDE10A1**  **normalized expression average** |
| --- | --- | --- | --- |
| 8 Putamen | 4.33 E-05 | 5.02 E-05 | 1.22 E-05 |
| 8 Caudate | 2.39 E-05 | 1.16 E-05 | 4.07 E-06 |
|  |  |  |  |
| **Relative expression results** | **Tukey post hoc test** |  |  |
| Putamen PDE10A19 vs PDE10A2 | p=0.8422 |  |  |
| Putamen PDE10A2 vs PDE10A1 | p=0.0150* |  |  |
| Putamen PDE10A19 vs PDE10A1 | p=0.0482* |  |  |
| Caudate PDE10A19 vs PDE10A2 | p=0.0087* |  |  |
| Caudate PDE10A2 vs PDE10A1 | p=0.1265 |  |  |
| Caudate PDE10A19 vs PDE10A1 | p=0.0010* |  |  |

**Table S6. PDE10A transcript abundance from RNAseq data of human striatal tissue.** The average of the normalized PDE10A transcript levels of all 8 individuals shown in Table 1 in both putamen and caudate nucleus tissues is presented here. A repeated measures ANOVA test was performed which indicated that there was a significant expression level difference between the three transcripts in both the putamen (p=0.0128) and caudate (p=0.0001) tissues. A Tukey’s post hoc test was performed to determine which transcripts were differentially expressed; significant differences are indicated with an asterisk.
