# Supplementary figures and images for: Novel, primate-specific PDE10A isoform highlights gene expression complexity in human striatum with implications on the molecular pathology of bipolar disorder
Source: Transl Psychiatry. 2016 Feb 23;6(2):e742–. doi: 10.1038/tp.2016.3 (PMC4872433; doi:10.1038/tp.2016.3)

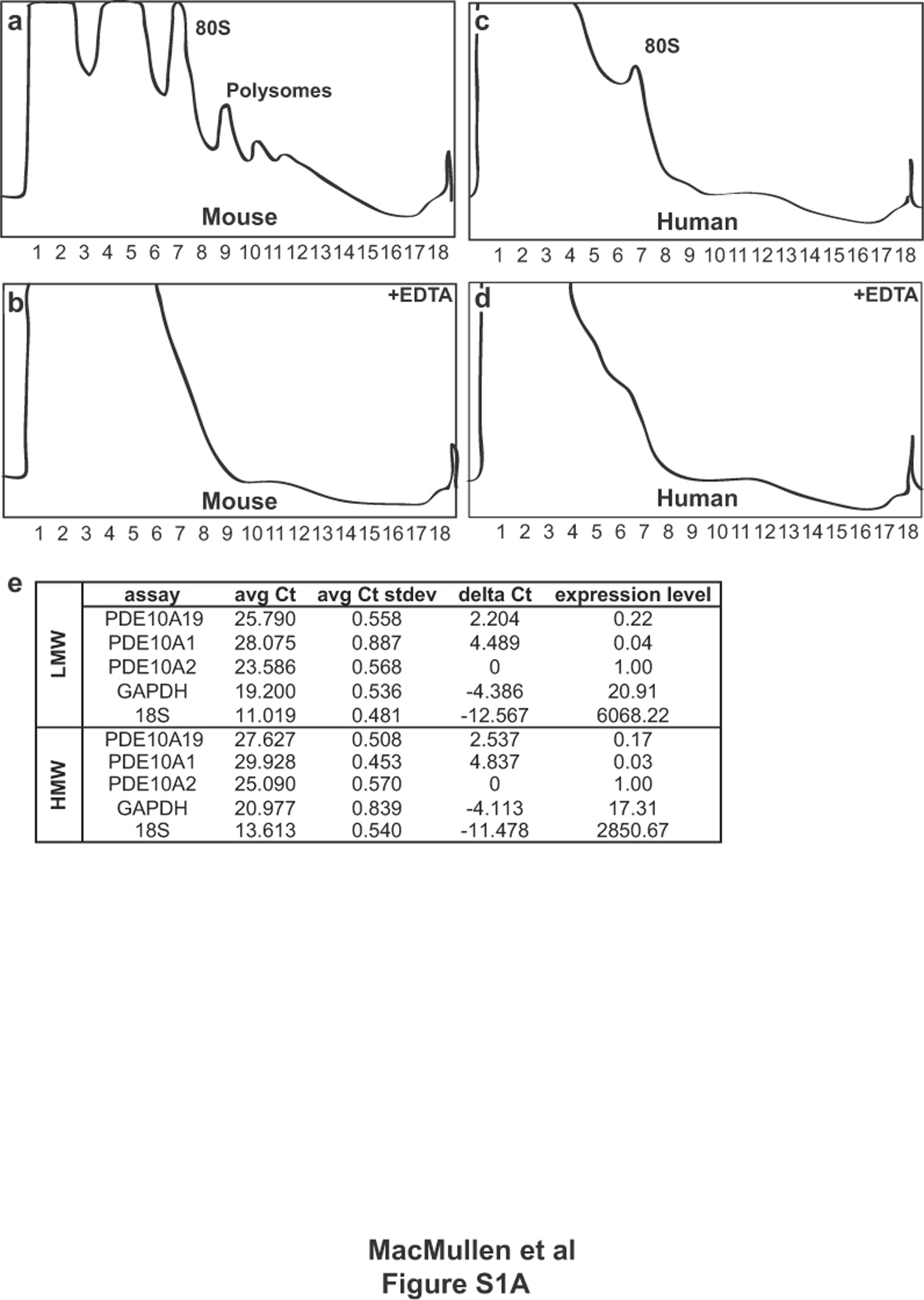

Supplement: Supplementary Figure S1a [file tp20163x8.tif]

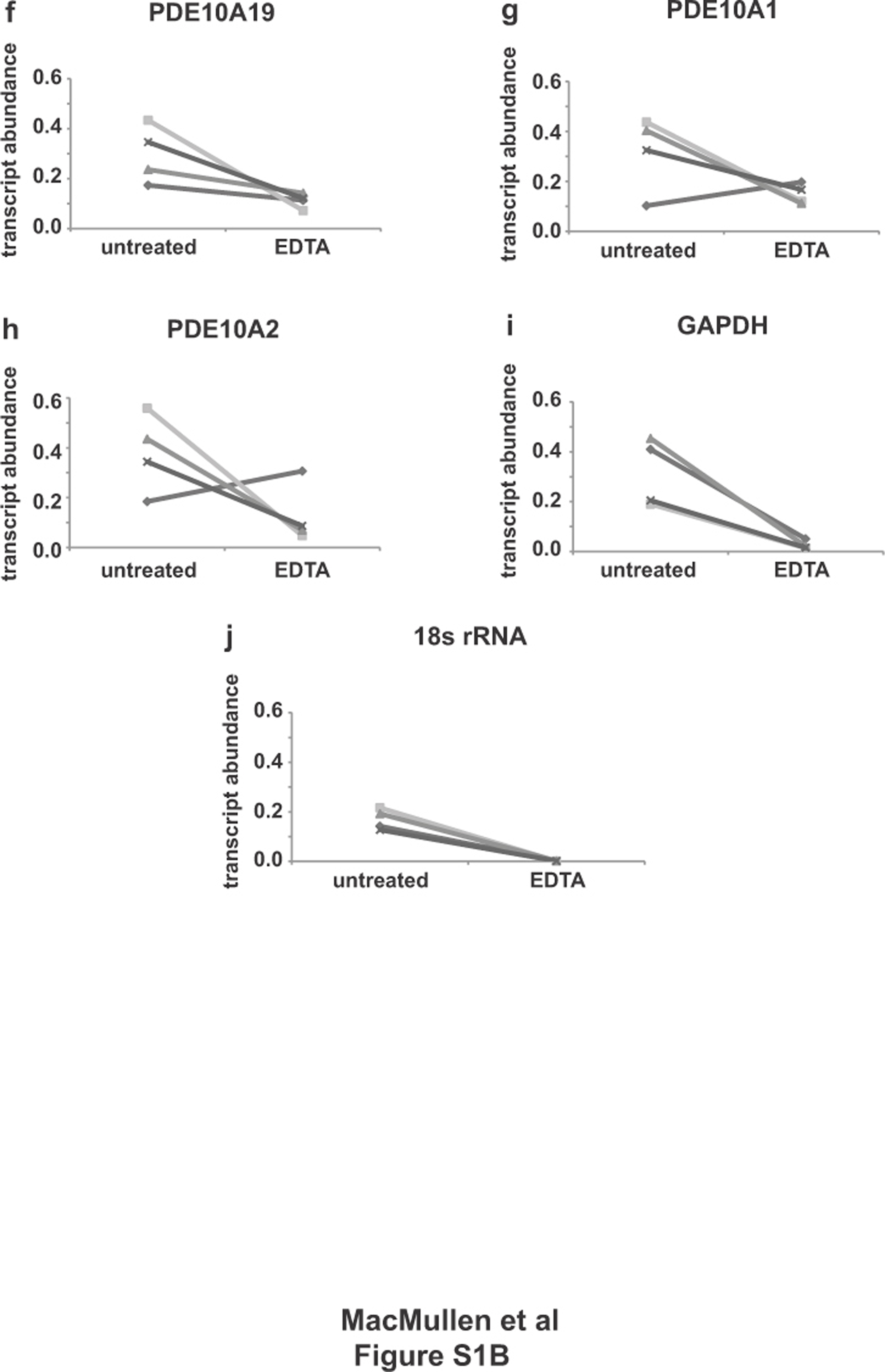

Supplement: Supplementary Figure S1b [file tp20163x9.tif]

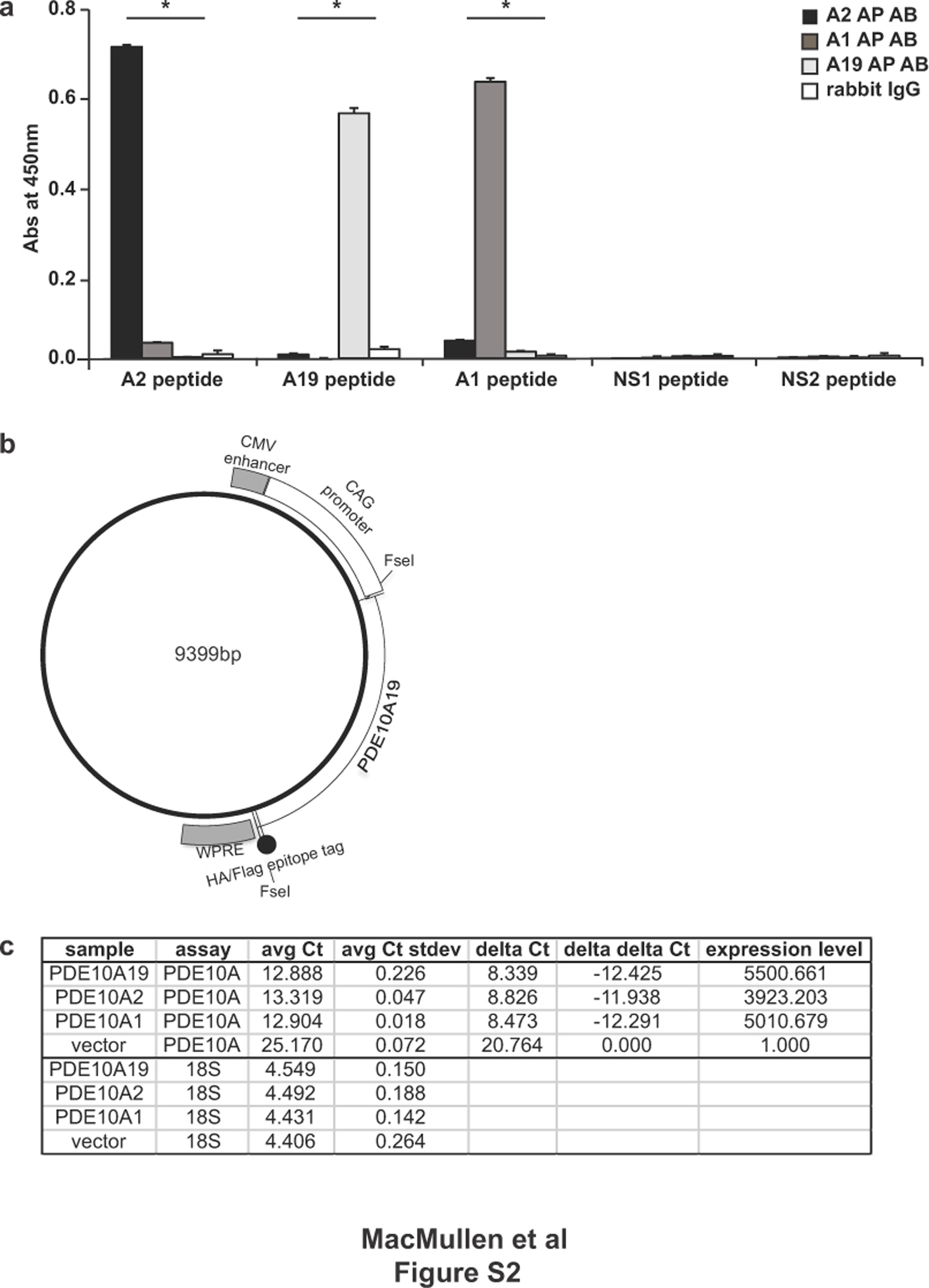

Supplement: Supplementary Figure S2 [file tp20163x10.tif]

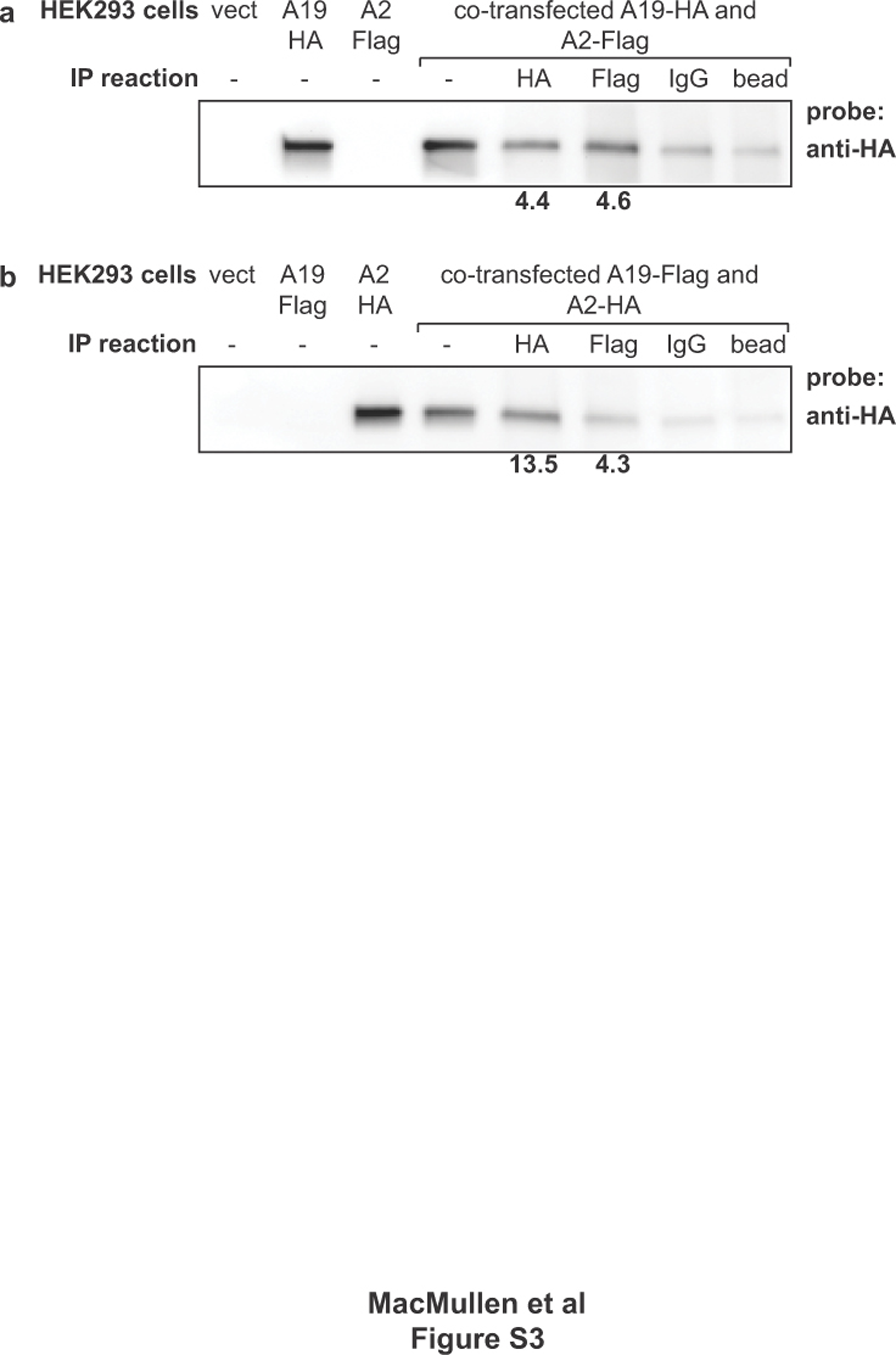

Supplement: Supplementary Figure S3 [file tp20163x11.tif]

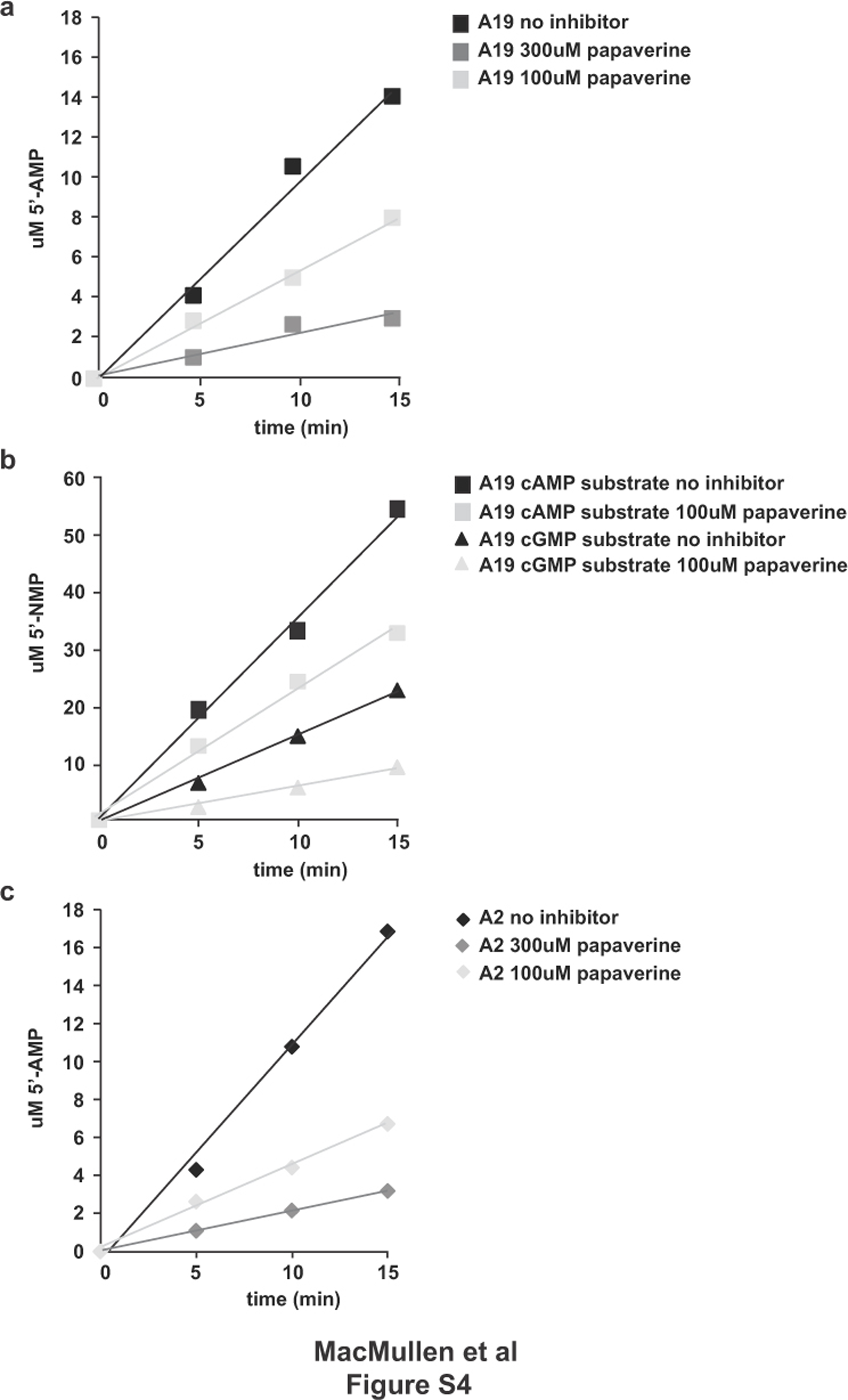

Supplement: Supplementary Figure S4 [file tp20163x12.tif]

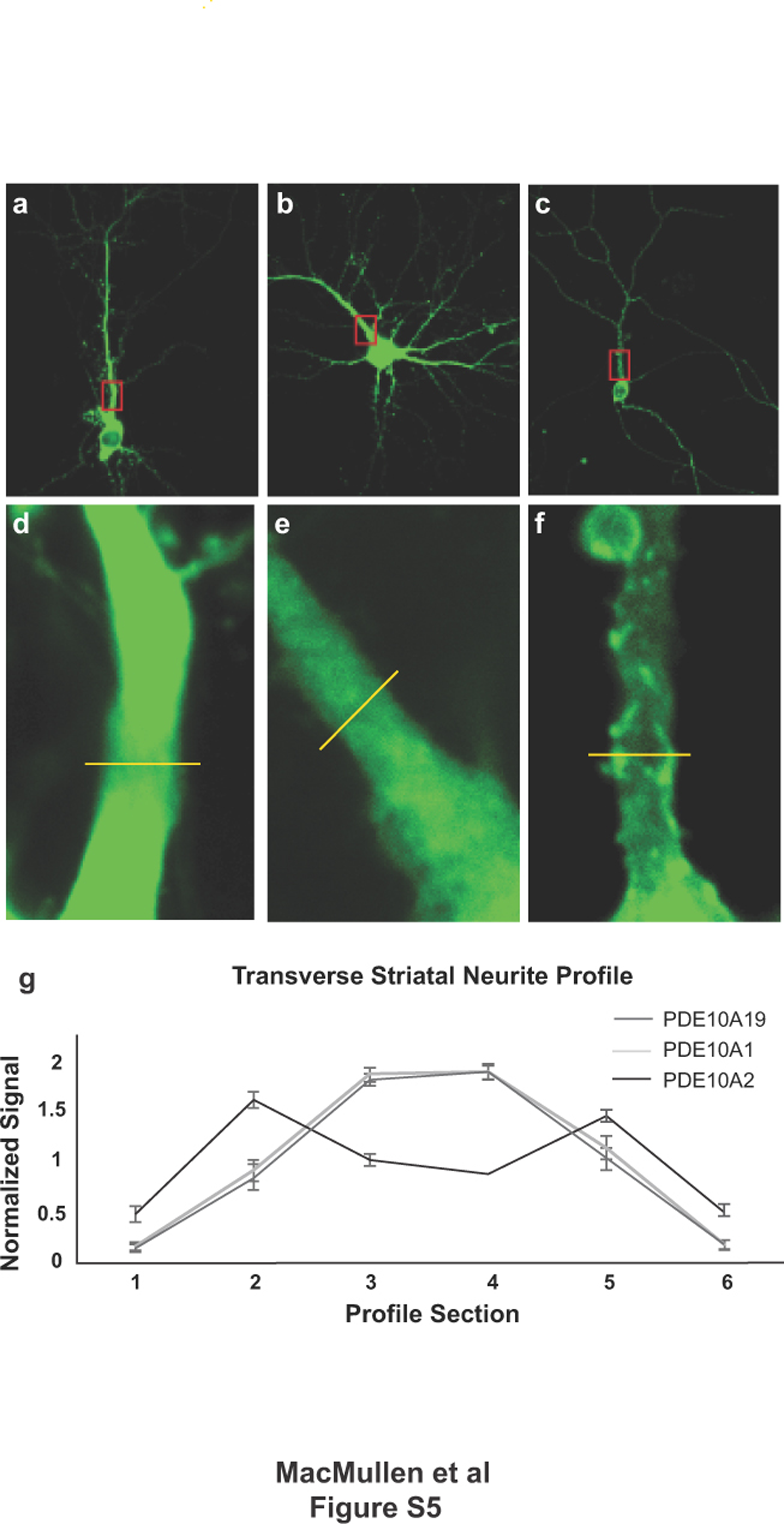

Supplement: Supplementary Figure S5 [file tp20163x13.tif]

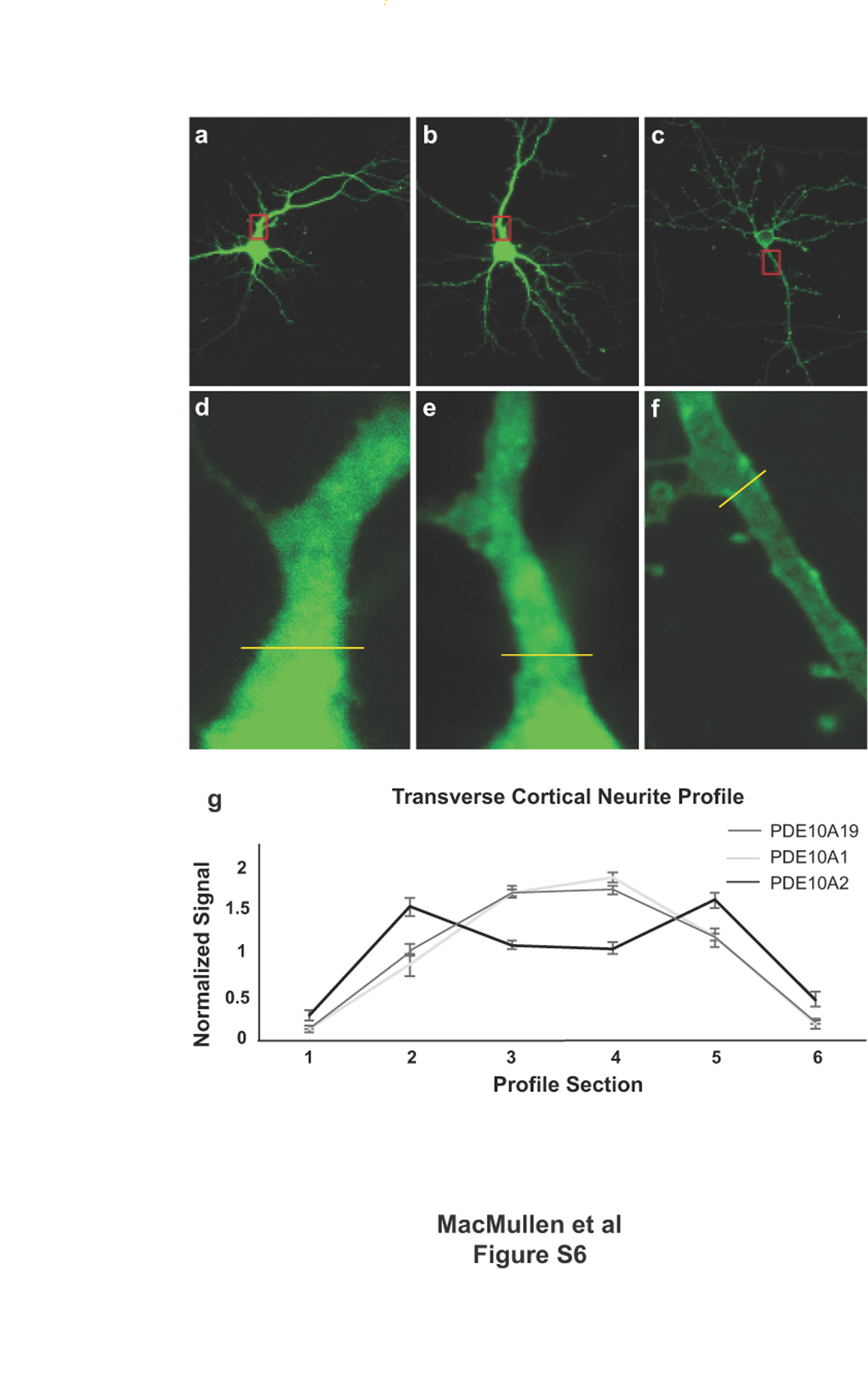

Supplement: Supplementary Figure S6 [file tp20163x14.tif]

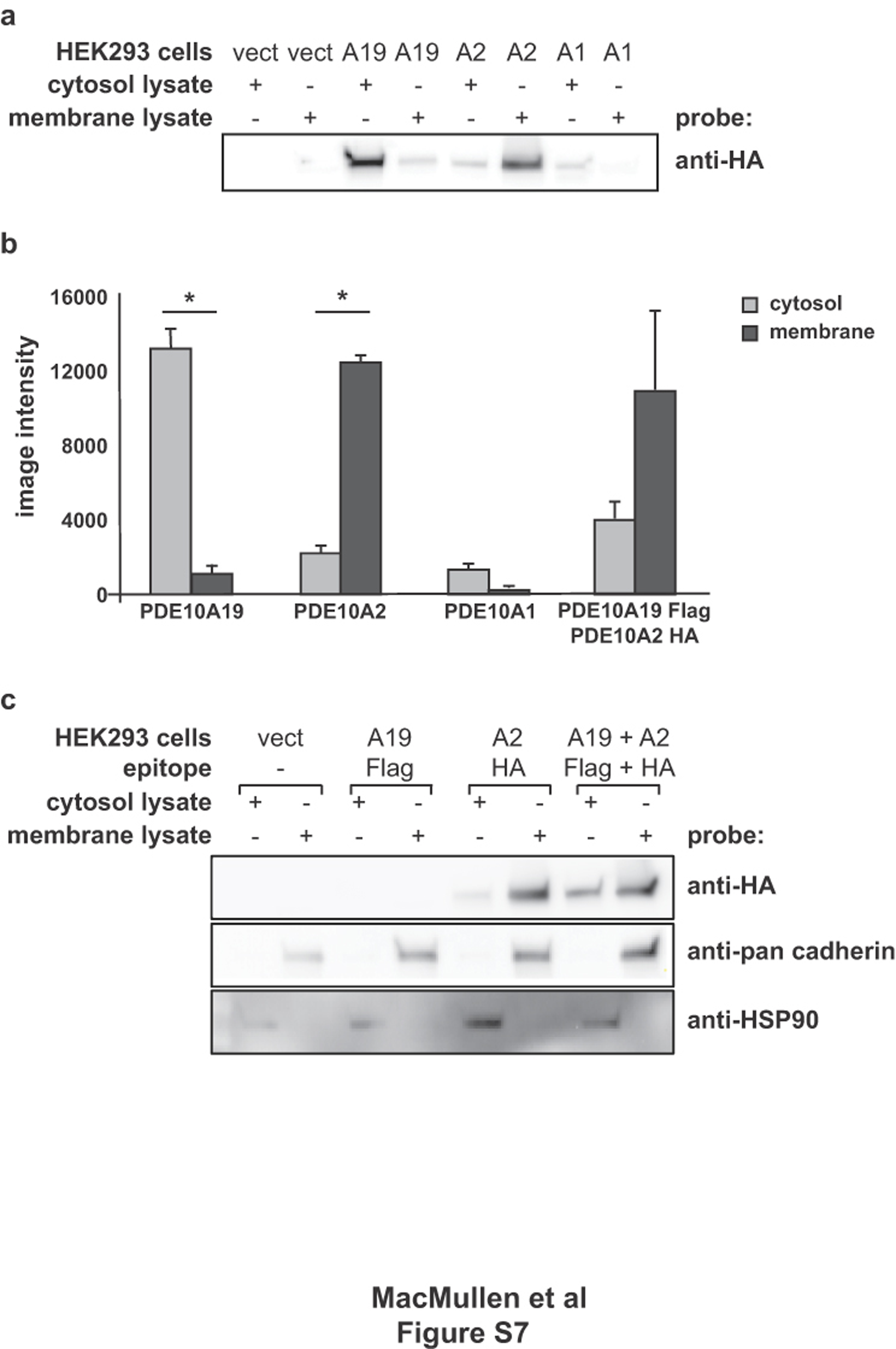

Supplement: Supplementary Figure S7 [file tp20163x15.tif]

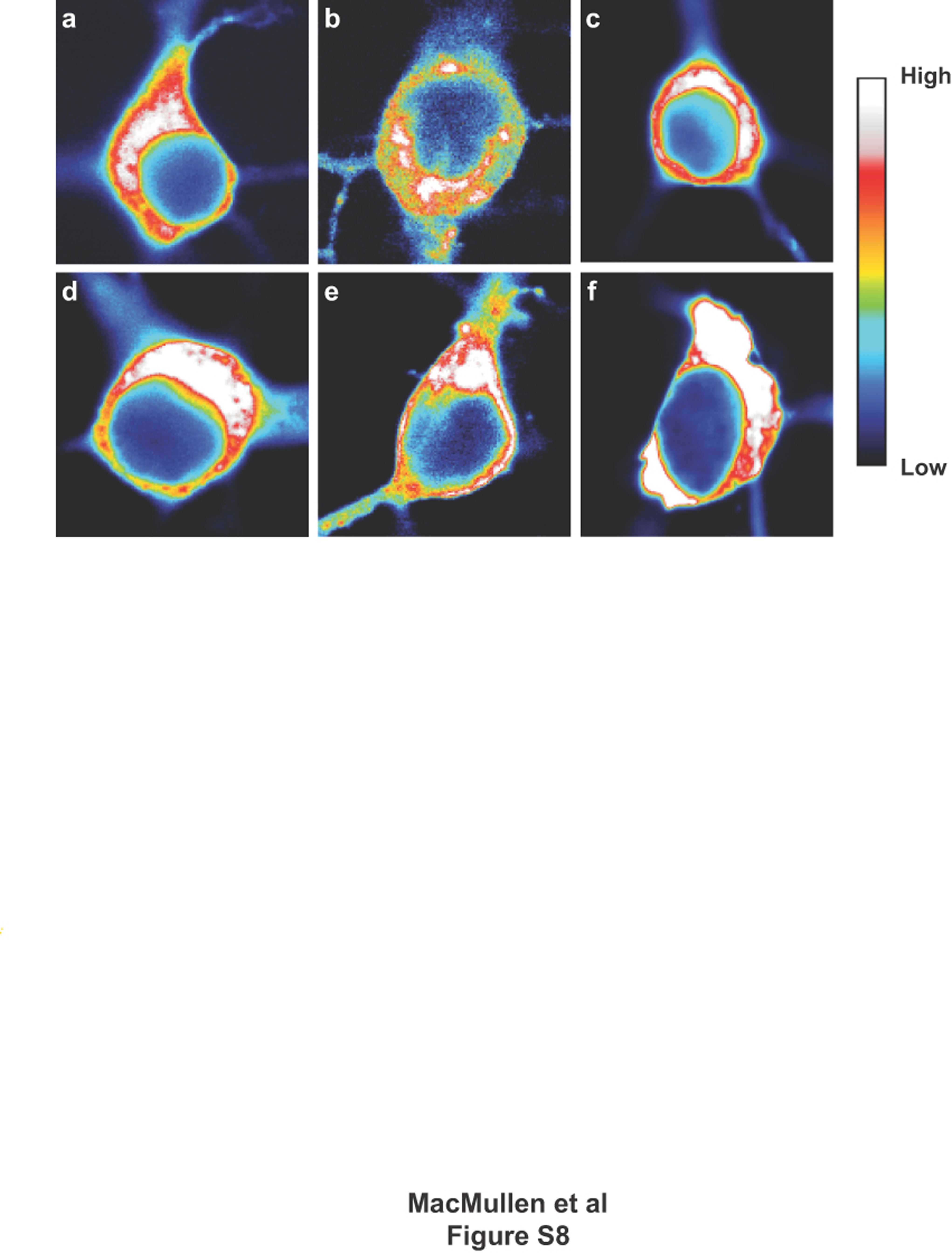

Supplement: Supplementary Figure S8 [file tp20163x16.tif]

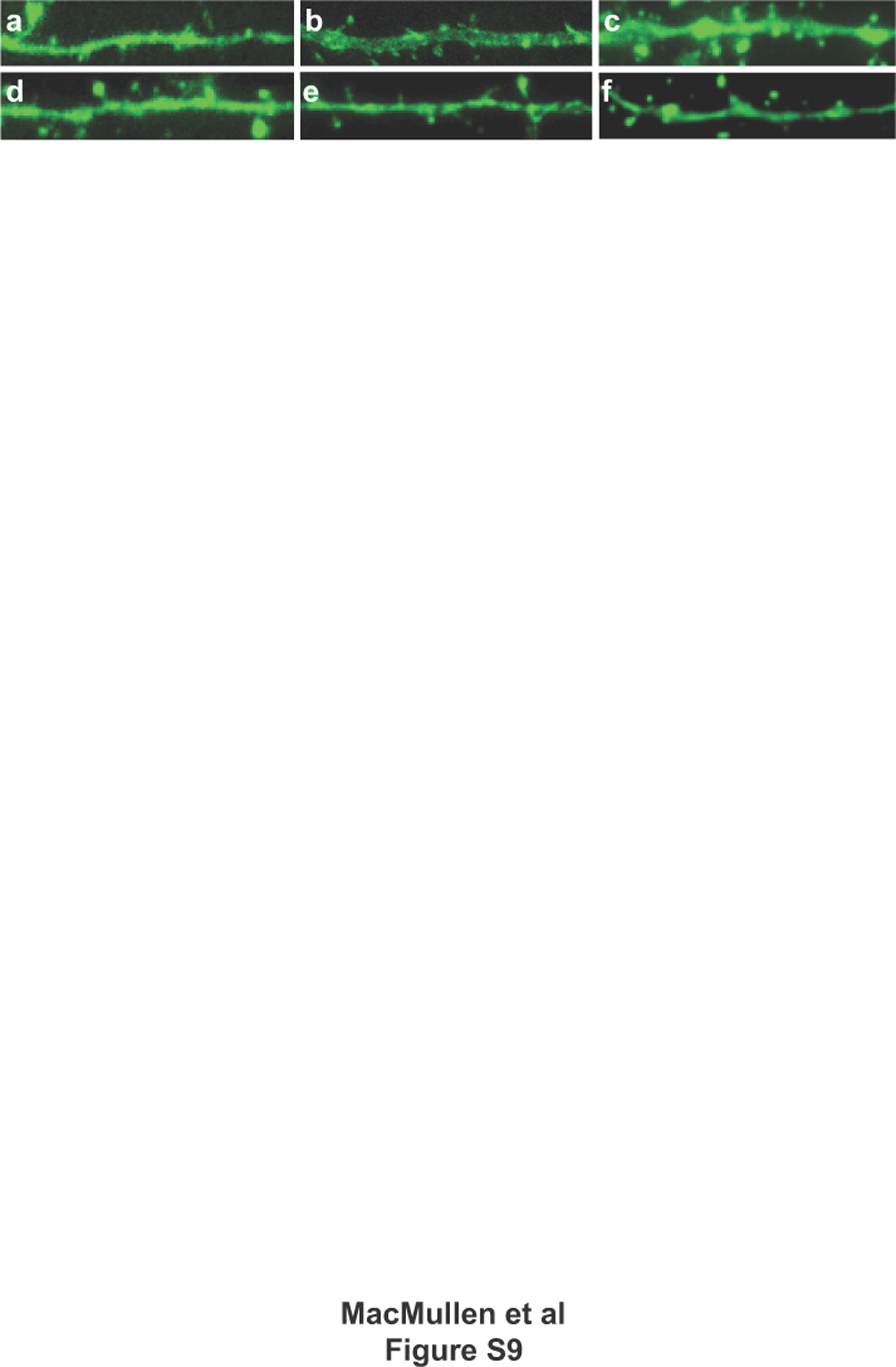

Supplement: Supplementary Figure S9 [file tp20163x17.tif]

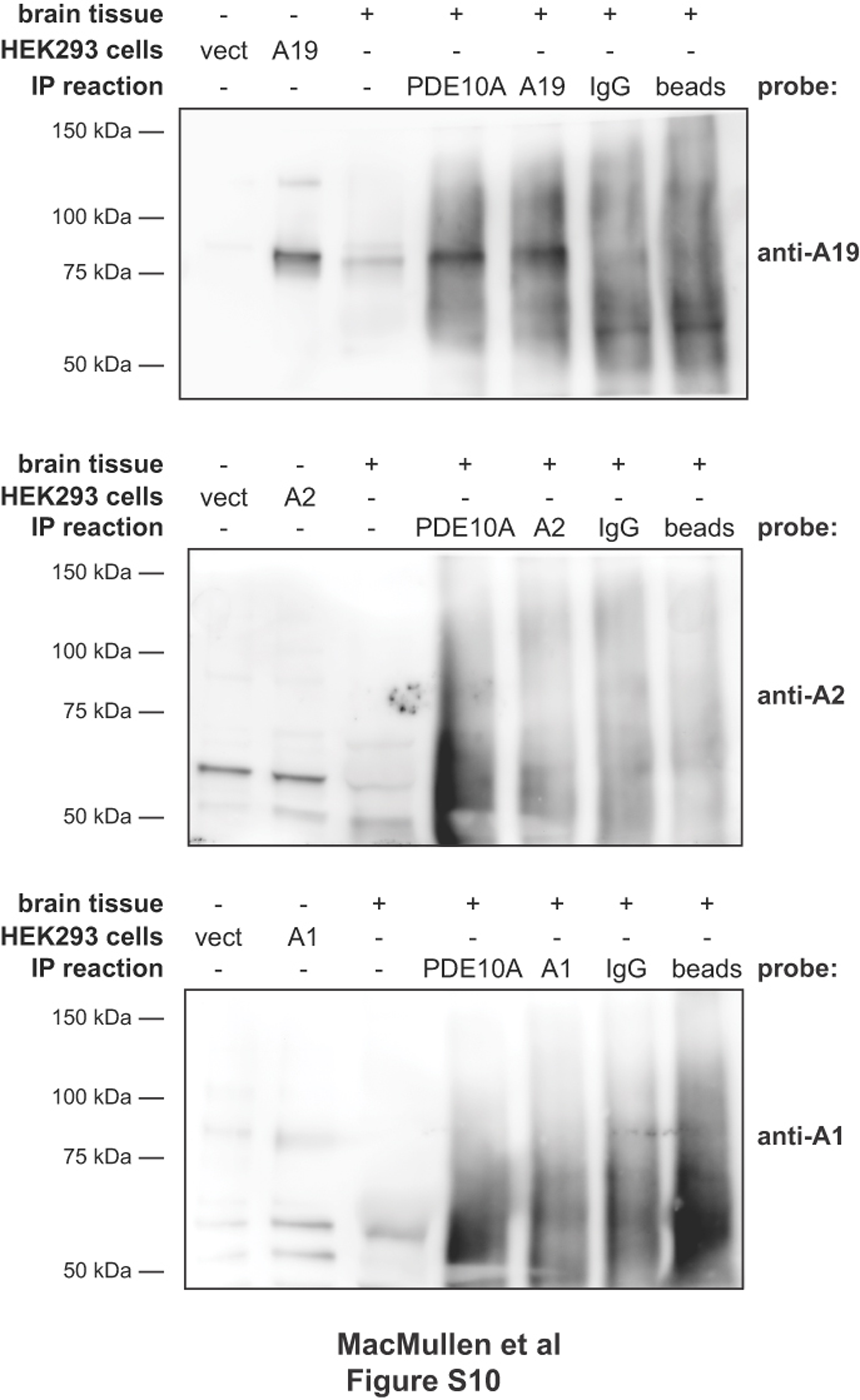

Supplement: Supplementary Figure S10 [file tp20163x18.tif]
