# Supplementary material for: Novel, primate-specific PDE10A isoform highlights gene expression complexity in human striatum with implications on the molecular pathology of bipolar disorder
Source: Transl Psychiatry. 2016 Feb 23;6(2):e742–. doi: 10.1038/tp.2016.3 (PMC4872433; doi:10.1038/tp.2016.3)
Supplement: Supplementary Figure Legends [file tp20163x19.docx]

**Supplementary Figure Legends**

**Figure S1. PDE10A RNA transcripts associate with polysomes in striatal tissue.** UV absorption profile (at 254 nm) of sucrose gradient fractions. Fresh mouse brain polysome preparations (**a**-**b**) were performed in parallel with human preparations to better identify fractions containing polysomes obtained from postmortem human tissue. Polysome peaks (fractions 9, 10, 11 and 12) are easily identifiable using mouse brain tissue (**a**); the polysome peaks are eliminated after EDTA treatment that destabilizes ribosomes (**b**). Although polysome peaks are not visible in postmortem human brain tissue (**c**-**d**), the 80S monosome peak was observed in untreated fractions and eliminated by EDTA (**d**). For each sample, fractions 1, 2, and 3 were combined as a low molecular weight (LMW) pool; fractions 9, 10, and 11 were combined as a high molecular weight (HMW) pool. (**e**) PDE10A transcripts associate with polysomes. Transcript expression levels relative to PDE10A2 were determined from the untreated preparations and were calculated as 2^-ΔCt^ (where ΔCt = Ct_target_ – Ct_PDE10A2_). The standard deviation illustrates the divergence of the four healthy control samples from the average Ct. (**f-j**) EDTA treatment can dissociate PDE10A transcripts from polysomes. PDE10A transcript relative abundances were measured in normal and EDTA-treated preparations for each of the four healthy control samples. The abundance of each transcript in the HMW pool relative to the LMW pool was calculated as 2^-ΔCt^ (where ΔCt = Ct_HMW_ – Ct_LMW_). The ribosome-destabilizing EDTA treatment caused a decrease in the amount of polysome-bound RNA (HMW) relative to free RNA (LMW). Although the effect of EDTA treatment was not statistically significant for the three PDE10A transcripts, possibly due to the low starting levels of polysome-bound RNA in postmortem tissue, a trend can be observed in most of the samples (p_GAPDH_ = 0.001, p_18S_ = 0.0001, p_PDE10A19_ = 0.057, p_PDE10A2_ = 0.137, p_PDE10A1_ = 0.253; paired Student’s t-test on mean ΔCt_normal_ vs. mean ΔCt_EDTA_; n=4 human samples).

**Figure S2. Custom anti-peptide antibody characterization.** (**a**) Indirect ELISA assay of custom anti-peptide antibodies. The three peptides used to generate the anti-peptide antibodies as well as two non-specific peptides to *D. melanogaster* proteins were coated on 96 well plates. Each custom anti-peptide antibody was allowed to bind to all five peptides and absorbance readings at 450nm were measured. ANOVA followed by Tukey’s post hoc test show that each anti-peptide antibody binds to its corresponding peptide significantly more than the other peptides tested (ANOVA p<0.0001 for all 3 anti-peptide antibodies tested). **(b)** Illustration of the pCAGWBA vector used to express HA/Flag-tagged PDE10A constructs. This vector was modified from pCDNA3.1, replacing the CMV promoter with the CAG promoter and adding a WPRE element for transcript stabilization. The HA or Flag epitope was placed in the open reading frame just upstream of the stop codon for each construct. (**c**) Quantitative data of qRT-PCR experiments for the three HA-tagged PDE10A constructs after transfection and expression in HEK293 cells. An 18S endogenous control was used to normalize the expression data. All proteins were expressed at 3000-5000 times over the empty vector alone.

**Figure S3. Immunoprecipitation of co-transfected PDE10A19 and PDE10A2 isoforms.** (**a**) HEK293 cells were co-transfected with HA-tagged PDE10A19 and Flag-tagged PDE10A2 isoforms. Cell lysates were immunoprecipitated with anti-HA (HA), anti-Flag (Flag) and anti-normal rabbit IgG (IgG), fractionated on western blots and probed with an anti-HA primary antibody. Image intensity values were taken for all IP lanes and the bead only control lane was subtracted from all other lanes as background. The anti-Flag image intensity was four times the anti-normal rabbit IgG IP indicating that PDE10A2 interacts with PDE10A19 (image intensity enrichment over IgG IP values are displayed beneath each IP reaction). (**b**) Reciprocal experiments were performed where the PDE10A19 isoform was Flag-tagged and the PDE10A2 isoform was HA-tagged. In this experiment, the anti-Flag IP image intensity was again four times the anti-normal rabbit IgG IP, confirming that the two isoforms interact irrespective of which epitope was used to tag the isoforms. Image intensity values were determined using ImageJ (NIH).

**Figure S4. PDE10A19 hydrolyzes both cAMP and cGMP.** HEK293 cells were transfected with HA-tagged PDE10A19 or HA-tagged PDE10A2 and the PDE10A19-HA and PDE10A2-HA enzymes were affinity purified. (**a**) PDE10A19 hydrolyzed an increasing amount of cAMP over time as measured by 5’-AMP production and was inhibited by papaverine, a known PDE10A specific inhibitor. This panel shows a representative result from three experiments performed with three independent enzyme purifications. Variability in transfection rates, expression obtained, and immunopurification efficiency between preparations prevent pooling the results from the three experiments to obtain group statistics. (**b**) When equivalent amounts of PDE10A19 enzyme were used (the same enzyme preparation), PDE10A19 was more efficient with cAMP than cGMP as substrate. Papaverine also inhibited the hydrolysis of cGMP. (**c**) PDE10A2 was immunopurified and used as a control, with this enzyme exhibiting linearity as a function of time and sensitivity to papaverine. The IC50 values of papaverine for PDE10A19 and PDE10A2 were virtually identical at 73.6 and 76.6 µM respectively. These values were derived from a plot of percent enzyme inhibition versus papaverine concentrations at 300 µM, 100 µM, 20 µM, and 1.7 µM. All graphs show the µM 5’-NMP product formed from reactions with 200 µM cNMP substrate.

**Figure S5. PDE10A protein localization in primary mouse striatal neurons.** Mouse striatal neurons were transfected with HA-tagged PDE10A19 (**a**,**d**), HA-tagged PDE10A1 (**b**,**e**), or HA-tagged PDE10A2 (**c**,**f**). Neurons were fixed and stained with anti-HA antibody conjugated to Alexa 488 and imaged. Red boxes in **a**-**c** represent the regions enlarged in **d**-**f**, respectively. Proximal neurites were selected and a line region of interest (ROI) was drawn across the neurites to quantitate expression (yellow lines). The ROI intensities of eight neurites for each condition were normalized, binned into six segments, averaged, and plotted with error bars representing standard error of the mean (SEM). (**g**) Both PDE10A19 and PDE10A1 display a single peak in the center of the histogram (sections 3 and 4) representing cytosolic localization. PDE10A2 displayed two intensity peaks at the edges of each plot (sections 2 and 5) representing membrane localization.

**Figure S6. PDE10A protein localization in primary mouse cortical neurons.** Mouse cortical neurons were transfected with HA-tagged PDE10A19 (**a**,**d**), HA-tagged PDE10A1 (**b**,**e**), or HA-tagged PDE10A2 (**c**,**f**). Neurons were fixed and stained with anti-HA antibody conjugated to Alexa 488 and imaged. Red boxes in **a**-**c** represent the regions enlarged in **d**-**f**, respectively. Proximal neurites were selected and a line region of interest (ROI) was drawn across the neurites to quantitate expression (yellow lines). The ROI intensities of eight neurites for each condition were normalized, binned into six segments, averaged, and plotted with error bars representing standard error of the mean (SEM). (**g**) Both PDE10A19 and PDE10A1 display a single peak in the center of the histogram (sections 3 and 4) representing cytosolic localization. PDE10A2 displayed two pixel intensity peaks at the edges of each plot (sections 2 and 5) representing membrane localization.

**Figure S7. PDE10A isoform localization in cytosolic and membrane fractions.** (**a**) HEK293 cells were transfected in triplicate with HA-tagged PDE10A19, HA-tagged PDE10A2, or HA-tagged PDE10A1. Proteins within the cytosolic fraction were separated from proteins within the membrane fraction from each transfection and the lysates fractionated on western blots to confirm the subcellular localization of each isoform. (**b**) The average image intensity and SEM of the biological replicates are graphed for each isoform for both the cytosolic and the membrane fractions. PDE10A19 was significantly enriched in the cytosol, while PDE10A2 was enriched in membrane fractions. PDE10A1 was enriched in the cytosolic fraction; however, the intensity difference between the two fractions was not statistically significant. The last set of bars represent the cytosolic vs membrane quantitation obtained from co-transfecting an HA-tagged PDE10A2 isoform with Flag-tagged PDE10A19 (see panel c). Asterisks show significant differences between cytosol and membrane band intensities determine by t-test. (**c**) HEK293 cells were transfected with Flag-tagged PDE10A19, HA-tagged PDE10A2, or co-transfected with both to determine whether expression of PDE10A19 alters the membrane localization of PDE10A2. Cytosolic and membrane protein fractions were fractionated on western blots and probed with anti-HA to detect PDE10A2, anti-pan-cadherin as a control for membrane localized proteins, and anti-HSP90 as a control for cytosolic localized proteins. Quantitation of the cytosol/membrane ratio for HA-tagged PDE10A2 from triplicate blots is illustrated in panel b (fourth set of bars). Although there was significant variability across the three transfections, PDE10A2 no longer retains its statistical enrichment in the membrane over cytosolic fraction.

**Figure S8. Nuclear exclusion of PDE10A isoform expression.** Mouse cortical (**a**-**c**) or striatal (**d**-**f**) neurons were transfected with HA-tagged PDE10A1 (**a**,**d**), HA-tagged PDE10A2 (**b**,**e**) or HA-tagged PDE10A19 (**c**,**f**). The neurons were fixed and stained with anti-HA antibody conjugated to Alexa 488 and imaged. A pseudocolor scale was used to depict PDE10A protein expression. In both cortical and striatal mouse neurons, the HA signal was excluded from the nucleus.

**Figure S9. PDE10A isoform expression in dendritic spines.** Mouse cortical (**a**-**c**) or striatal (**d**-**f**) neurons were transfected with HA-tagged PDE10A1 (**a, d**), HA-tagged PDE10A2 (**b, e**) or HA-tagged PDE10A19 (**c, f**). The neurons were fixed and stained with anti-HA antibody conjugated to Alexa 488 and imaged. In both cortical and striatal mouse neurons, significant HA signal was localized to dendritic spine heads for each of the HA-tagged PDE10A isoforms.

**Figure S10. PDE10A19 is expressed in human striatum**. Full-length blots of those illustrated in Figure 3c. Human striatal tissue from HC 3589 was used for IP reactions with a commercially available PDE10A antibody (PDE10A-carboxy) that recognizes the carboxy termini of PDE10A isoforms. Isoform-specific, N-terminal anti-peptide antibodies for PDE10A19, PDE10A2, and PDE10A1 were also used for immunoprecipitation and to probe the blots. An immunoreactive protein of the same apparent mass as detected in transfected HEK293 cells was detected in striatal immunoprecipitates using the PDE10A-carboxy and PDE10A19 anti-peptide antibodies. No immunoreactivity was observed in any of the lanes using the anti-PDE10A2 anti-peptide antibody. This result was expected based on prior results using transfected HEK293 cell lysates . No immunoreactivity was observed in any of the lanes except the HEK293 cell control lane using the anti-PDE10A1 anti-peptide antibody, presumably due to its low abundance of expression. Abbreviations for HEK293 cells: A19 = cells expressing the novel HA-tagged PDE10A19; A2 = cells expressing HA-tagged PDE10A2; A1 = cells expressing HA-tagged PDE10A1; vect = cells transfected with the empty vector; untrns = untransfected control cells. Abbreviations for IP reactions: PDE10A = PDE10A-carboxy; A19 = PDE10A19 specific anti-peptide antibody; A2 = PDE10A2 specific anti-peptide antibody; A1 = PDE10A1 specific anti-peptide antibody; IgG = normal rabbit IgG antibody; beads = no primary antibody was used for IP, only Protein G Plus/Protein A beads were used for purification. Abbreviations for brain tissue: “+” indicates lanes where brain tissue lysates were added.
