# Supplementary material for: Novel, primate-specific PDE10A isoform highlights gene expression complexity in human striatum with implications on the molecular pathology of bipolar disorder
Source: Transl Psychiatry. 2016 Feb 23;6(2):e742–. doi: 10.1038/tp.2016.3 (PMC4872433; doi:10.1038/tp.2016.3)
Supplement: Supplementary File 1 [file tp20163x20.docx]

PDE10A19 transcript sequence, genbank accession number KU232567 (ATG start site and TGA stop codon are bolded and underlined):

GCCTGAGATGCATATGCTAGGAAGGCAGTTGGATAAACAAAGCCTGGGAGGATTTGATCCAAAGGATCGTTCAAGACTGCAGCATAGAAACTGCAGTCACAGCATCTAAAATCATGGGATTGGATGCCCCCAAGGACAGTATATAGATGCCTCAGTGACATTTTGACTCTGAGGGAAATACAGACGGAGTTGGTTAATCAACCATAGCTGGGTGCAATGTAAGTGCCTGGCTGAAGTTTGACACGCGAACGGACGGCCCGCTGGAATTCTGTGCTATGAGCCGGAGTAGAAAGAGAGATTTGGACTCTGCAACACCAAGGTAGTCGTTGAAGCCACAGTCGTGAATGGAGACCAGGAGTGAATAGTGGGAGTGAGCAGAAGTCGGAGGATAGGACAGAAGAAGGCAGAGCCATGGAGCACCCTGGAGAGGTGTGACCCGGCAAGATCCTGAG**ATG**GAAGGTAGCACGGCCTGGAGTTCAGAAGCGGAGCCTCAAGAGGGAAGAAGCCAGATGCTCCAGAGAGCAGGTTTGACAGATGAAAAAGTGAAGGCATATCTTTCTCTTCACCCCCAGGTATTAGATGAATTTGTATCTGAAAGTGTTAGTGCAGAGACAGTAGAGAAATGGCTGAAGAGGAAGAACAACAAATCAGAAGATGAATCGGCTCCTAAGGAAGTCAGCAGGTACCAAGATACGAATATGCAGGGAGTTGTATATGAACTAAACAGCTATATAGAACAACGGTTGGACACAGGAGGAGACAACCAGCTACTCCTCTATGAACTGAGCAGCATCATTAAAATAGCCACAAAAGCCGATGGATTTGCACTGTATTTCCTTGGAGAGTGCAATAATAGCCTGTGTATATTCACGCCACCTGGGATAAAGGAAGGAAAACCCCGCCTCATCCCTGCTGGGCCCATCACTCAGGGCACCACCGTCTCTGCTTATGTGGCCAAGTCCAGGAAAACACTGCTAGTAGAAGACATCCTTGGAGATGAACGATTTCCAAGAGGTACTGGACTGGAATCAGGGACTCGTATCCAGTCTGTTCTTTGCTTACCAATTGTCACTGCAATTGGTGACTTGATTGGTATTCTCGAGCTGTATCGGCACTGGGGCAAAGAAGCCTTCTGTCTTAGTCACCAGGAGGTTGCAACAGCAAATCTTGCCTGGGCTTCAGTAGCAATACATCAGGTGCAGGTATGCAGAGGCCTTGCCAAACAGACAGAATTGAATGACTTCCTACTCGACGTATCAAAAACATATTTTGATAACATAGTTGCAATAGATTCTCTACTTGAACACATAATGATATATGCAAAAAACCTGGTGAATGCCGATCGTTGTGCGCTTTTCCAGGTGGACCATAAGAACAAGGAGTTATATTCAGACCTTTTTGATATTGGAGAGGAAAAGGAAGGAAAACCTGTCTTCAAGAAGACCAAAGAGATAAGATTTTCAATTGAGAAAGGAATTGCTGGCCAAGTAGCAAGAACAGGGGAAGTCCTGAACATTCCAGATGCCTATGCAGACCCACGCTTTAACAGAGAAGTAGACTTGTACACAGGCTACACCACGCGGAACATCCTGTGCATGCCCATCGTCAGCCGAGGCAGCGTGATAGGTGTGGTGCAGATGGTCAACAAAATCAGTGGCAGTGCCTTCTCTAAAACAGATGAAAACAACTTCAAAATGTTTGCCGTCTTTTGTGCTTTAGCCTTACACTGTGCTAATATGTATCATAGAATTCGCCACTCAGAGTGCATTTACCGGGTAACGATGGAAAAGCTGTCCTACCATAGCATTTGTACTTCAGAAGAGTGGCAAGGTCTCATGCAATTCACCCTTCCCGTGCGTCTCTGCAAAGAAATTGAATTATTCCACTTTGACATTGGTCCTTTTGAAAACATGTGGCCTGGAATTTTTGTCTACATGGTTCATCGGTCCTGTGGGACATCCTGCTTTGAGCTTGAAAAGTTGTGTCGTTTTATTATGTCTGTGAAGAAGAACTATCGGCGGGTTCCTTATCACAACTGGAAGCATGCGGTCACTGTAGCACACTGCATGTATGCCATACTTCAGAACAATCACACGCTTTTCACAGACCTTGAGCGCAAAGGACTGCTGATTGCGTGTCTGTGTCATGACCTGGACCACAGGGGCTTCAGTAACAGCTACCTGCAGAAGTTCGACCACCCTCTGGCCGCTCTCTACTCCACTTCCACCATGGAGCAGCACCACTTCTCCCAGACTGTGTCCATCCTCCAGTTGGAAGGGCACAATATCTTCTCCACTCTGAGCTCCAGTGAATATGAGCAGGTGCTTGAGATCATCCGCAAAGCCATCATTGCCACAGACCTTGCTTTATACTTTGGAAACAGGAAGCAGTTGGAAGAGATGTACCAGACCGGATCACTAAACCTTAATAATCAATCACATAGAGACCGTGTAATTGGTTTGATGATGACTGCCTGTGACCTTTGTTCTGTGACAAAACTGTGGCCCGTTACAAAATTGACGGCAAATGATATATATGCAGAATTCTGGGCTGAGGGTGATGAAATGAAGAAATTGGGAATACAGCCTATTCCTATGATGGACAGAGACAAGAAGGATGAAGTCCCCCAAGGCCAGCTTGGGTTCTACAATGCCGTGGCCATTCCCTGCTATACAACCCTTACCCAGATCCTCCCTCCCACGGAGCCTCTTCTGAAAGCATGCAGGGATAATCTCAGTCAGTGGGAGAAGGTGATTCGAGGGGAGGAGACTGCAACCTGGATTTCATCCCCATCCGTGGCTCAGAAGGCAGCTGCATCTGAAGAT**TGA**GCACTGGTCACCCTGACACGCTGTCCCACCTACAGATCCTCATCTTGCTTCTTTGACATTCTTTTCCTTTTTTTGGGGGGGGTGGGGGGAACCTGCACCTGGTAACTGGGGTGCAAACCTCTTCAAGAAGGTAACATCAAATAAATAAGTCAAGCAGAGGACTTCCTGCCAATCTCTTCTGTGAGGCATCATAGACACTGAGCAACCAGGACCACCCCCACGTTCAGAAATCAGCTGGCCAAGTGACTCCATTTGACTTGCAAACCAGCCTTTTCTAATAGGCTAATATTGCTGAGGCCTTAAAGGAAATGGACAAAAATTATCCAGAAGGGGTACTTTTCCATTGTATCTTTCTAATAAGGGTTTAAAATGGTACTATTATGGTATTGTACTTGGGCTTTAACATCAATGTTGCTTTGATGTTGTTGGATATAAATAGGAATTTTTACACATTACTATTGTGAATGGTGAATGTTCATGTATGACCTACTTGTAATTAACTTGAGTTGTAGTCCACAGCCTCAGGACAAATGTCGTTGAGGTTACAGAGTAAGAAATGATGGCAAAACGTCAAACTCTTATTTCAGAGCTTCATGAATTTAGTTAGACTAAACATAATTCTTTAAGTTCAACCTAAAGGGCTGAGATCAATAAATTTAACACTAGACGAAGTAGACTTCCTGTCTTTTTGAGAAGAGATGAGGTATATGTTACAATAAATCTCAGAACTTCAAGTAGCAGTTCAAAAGATGTCAGTTTTTAAAATTGTTTTTGTTGTTGTCTTGGCAGTTTTACTGAACCCTTTGCATAAAGAACAAAATAAAAGCTCGGCATTGTAATTTTTTTAATGGACAAGTCTTATGGATACGAAGGGTACATTTTTCATAATGATTCCTTTATATTTTCACTTTGTGTCATTGCAGAATTTTAGACTCTCATTCACAATGAAAAGTTTATTTTAAACATTGTTTAATTAAAATACCATACAGTTCTCTTTTAAACATCAAACCATAAAAAGTGTATTTTGTAATTTTACTCTGACCTGCCGCAGTCACCTCTCACTTATCTCTTCCACGTACTGCACGGTCGTATTTCATGAGCTTTCTGTCCATAGCACAGAAACAGAGCAGAAAGTAGTACAATCATGTTGGACCTTCTTTCTGTTCTCTTTACTCTTCTCACAGATCAGATCACTCCATAGAAGCCTGTGGGTTTCGATGGTTTCTTCTATACACCTTTTTGGTTGACCAGTATTACTATACAATGTAAGTGTTTTAAAAAATACGAAAGTAATACTCTGCACCCCTTCCTACAAAGATGATAAAGCAGTCACTTCTGGCGCATTTTAATAATTTAAAGATTTTTAGTGCAATGGCACGGTAACCTCCAAACCTGAATTAGACAGAGACTCACTCAGGAAGTGACAGGCCCATCATATCAAATAACTTATTCACTTTTCATGTGGCAGGAAACTGGAATATCGCTTTTAATAAAATGGAAAAATATGCTTCTACATATTTACCACCATAGGCGTTTTGTTCATATGAGCCTGGTTTGTGCAAAATTAAATCAGAGGCTTCTACAACATGGTTTATTTATGTTGTAGCAAAGTTGGCTCTACATAAACATTGTTCTTATTTTAAAATTAACACTATGTGTTCAGTTTTCTTGTGGGCTTCTGAAAGTTGCCATCTTCCCTCCGTGGAGCTCCATTTGCTATTTTCATTATACACTATGAGGTAAAATGTAATAACAAAAGAGAGAGAAGTACCACTGTGGCTAGATATATACACACACATATATATATGGATGGATGTAATATATGTAGAACACACACATAGATGTATATAGGATACACACTCATGTATGTAAACGTATACATATGTGTATATATGATACATACACATACACACACACGAGAGACAGAAGGAAAGAGAGGAAGAGAGAAGCAAACATGTAGGAAAAAATATAAATCAGCCCCATTCTCCCATTCTTTAAGAACAATGATTTTTCTTGATGGAAAATGCATATCACTTGACAGTTGACAAGAAAGTGTCATTTTTGTGTTAGTGTATCATATAATTGAATTTGAACAAGATACATATTTTTCTGCATACAAATTTATGAAGCTGGAGTCTACCAAACACAGCTTTAGTCACAAAAGTTAGTTTAGTTATCTCATTTCGCTTGGAGATGGAATTTCAGGAAGGGATATATTATTTAAGATTATTGTTTAATTACAAGAATTTTAAAAGAATGTATGAATTCTGTTTTTTAATATAGAAAATAATTATGGAGTTAGGAATCTCATGGCTACATTTACATTTTTAGGTTATTACAGAAAATTCTCTTAATTTATTATAGTCAAGTGTATTATGTAATCTCTATTTAGAACAGTTTCCCAAAGGAATGTGATTGGATTTACTGGTTGGTTAATTTCTTGTGAAATATTGTCATAATAGAAGCACAGCTTGAACAAAAGACTGAATTCTGATATAAACTGTCAATATTGCTTTTACCATGTGTAAGATTTACTTAGTAATCACAGATTTACCTAAAATAAAAGGACCCGGGTTGCAGTGATATTTAGTTAATGATTTGTTTTGGAAAACATGCAGTTAGGAGTATTTTTGTATTTTGTCAGATGTTTAGGAGACAGACTTTCGGGTCATGTTTCTTGTCTTGTTCCCAGATCTTTTCTTAAAAGAACAGTGTGACACCAGAAGCCAAGAGATGGTTTTTACTAACAACAGCTTTGATGAGTTCCTATTAAGGTCGTATTGTGTATTGTGCCAAATTTTCCACCCAGTATAAATTTAGTAACCCGCTATGAGCATATGTAGGATATTCAATTTGCAGATACTTTTGTTGGTTATGTTGGTTTATAATAGACAAGTTTTATGGTCATATGCAATTATTCCATTATCTTTTTGGACACAGTGAATATGTTTTTTAGCTAAAAATGTCCTTGGAAACATAAATGCCTGCCAATAACATGGAATATTGAATTCGGGCAATGTGAATTGATTAATTAGAAAAGGGTCAAAAGAAATACAATGGATGTGGAAACTAATTCTAAAAAATAGCCCTATTAATAGGGCAGAGACTCTGGTCCATTTCCCAGCTGTACTTCTCTCTAAGAGGTGTATGAAAAACCCATCTTGCAAAAGAGACCAAAATATTAGACACAATCGTTCCCATTCCTGACTCTTCAATGGAAAAGGAAGGTACAGATGTTTACAGTGGTGCCCCAGGTGACTCGTTCTCACCACAACAAAAGCAACTAATTGGAAACTAGATGTGTTCAATAATAGATTTCCTCTTTTTTGGCATGCTTTTTAGATTTTTTTTCATTTTGTAAAATCTCAGTTAATTTTTCTTTATTGGTATATTTGTGTATGCGAGACAAATCCTGTATTGTACAGGATATAAATAGATGCATAAATCCTTAGATACCTGGGTTTTTTAATAGGTTTAGGTTTGGGTATTGTGTTTCTGGTCTTTTAACTCAAAATTAAGGTTTGAGCTTTAATAGGACTAAATTATTCACCTGCCTTGGCCTGTGCCTTTATTTTAAATACTGCAATTTTACTTCTTCATTCATGTATTTATTATTTTGCTTTTCTTGCTTAAGTATAATTGAAAATATTTGCAGGAACGCACAATTCATTTTATCAAGTTGAATGAATAGGACCATTCTCTCCTCATCAGATTTCTGTTCTTTATTTGACTTCATTGACATATACCCTGGTGACTATAACCAGTGAACTGTTCAGAGTCAGTCTTTTCGGCAAGAACTTCAGAGAACTGTGACACTGGTTATGATGTGATTTTGGGGGGTTTCAGGTACCTAAGCATGATTTTTAAAATCACTTCTCTCTTTACATATTTCGAACATATGTTTGCTTTCTACACTTTGCAGATTTTATTTAGGATTTAATTCTTTAAAGACTATATTCATTTAGTCACTATAGTGGATCTCTTCTACTGCAAGTATTCATCTGATTTTTAAATTATGTAAAATGTAAATCAGCCAAGTATGTCATCCGTTATAGTTTCCCCATGCTTTCAGCGCATGGCCATGAGATTTTTACATGTGCATAAGTATTTAGCCAATACACATTTTGCTGACTTCTTTAACCATTTATATTGTTTAAATTAGTAATATCCAATGCAGTGCACACTTTTTTGTCTTTTTTCCAACGGAATATTTTGGTTGTCTGTAGCGTTGGAAGCATTTTATATGATAAAAATAGCAAGAAAAAATTAAAAAGTGTCAACGGTGTGCTGGTTCTAAATTAAGAAGATAGAACAAAGAGAAAGACATTAATGTTACCTAAGAAACACATTTAACATCCGAAGAAGCAATGCTGTCCTCACTTGATGTTTCTCTTCTCAACCCAGCAATGGAGAGCAGGGCTTGCAAAGCCGTACAGAGCCTACGTGCTCTTTCCTGCAAGTGGGAGAAAACTAATCAATGGCACACAGGGCCAGGTGTGGCCCTGTGAGGCAAAGGATAATGTCACCTCAGGACCAAGGTGGAAACTTACAGCTGAATCCAGGGCTTACTAAGTAGGGAAGTCAGTTTACATATTTCAGTTTTCCTCTGATGTTTTTGAGGTACTGTGTATTTGCAGAAGTTTAAGCAGAAGGGTTTTGTTTCTATTGTAAGTTAGGTTCTAAAAGTGTATAATCTGATACTCTAGCCCAACTGAAATATTATATCTTGGGCGTTACAGCCTTTGTCTTTTTCTTTAGTCACCTTAATACCCAGTCCTATGCTGTATCTCTGATAGCCTATATAAATGACATATAGGAATATTATTTTCCATATGATCTGTTGTATGGTGTATATGCTATGTGTTCATTTCGTGTGCAAGTTCAGAGTGTAATAGATTTACCCAGAGAAGGTGTTTCTATACAATGCCCCTACCTATTTGAAATTACATGCTTGCCCAACACACTGTGAAATAGTTACCAAAATTTGTACAAATGCAGCATCTTCATTCTTTCTGAGAAGACAAGATGGTTTTCTTTACATGAACAAATGAACAAAAGAGATCCTAGATCCATAACGTAGCTAAGGCATCTAAGAGTTTGCTGTTGATAATCTTGCTGACCAAAAACTACTGGAGAGTAACACAGGTTATATGCCATCACAAATACAATGCTCATGAAGAACTGATTTGTAGAGTCAATGAACCTGTGTCCAGAATTTTAATAGGCTCTCTATTGGAAGGAGAAAGAATTTCAAGTTAACAGTATCTAACTTTATCATAGTTGATGTTAGTAAATTTTAAAAAATGATTTTATATGTATGACAAAAATCTTTGTAAAATGCGCAAGTGCAATAATTTAAAGAGGTCTTAACTTTGCATTTATAAATTATAAATATTGTACATGTGTGTAATTTTTTCATGTATTCATTTGCAGTCTTTGTATTTAAAAAACATTTACTGTTATGTTTGTATAATAGAACAGTAATCATTTATTATAATCTAGGCAAGTTGTAAATAAATTCATAATTCAAACAGCCAGTATATATGCATATATGAGTGTTATATTGCAAAATCTTTGTTTTACTTACATGGTTAAAGCAGCAAGAATTCTTTTGTTGATATGTAATTATACACATAAAATATATATATGTATGATACATGAAATATATTTAGAAATGTTCATAATTTTAATGGATATCCTTTGGTGTGAATAATTGAATACAGAGTTTTTAAAATA

PDE10A19 protein sequence:

MEGSTAWSSEAEPQEGRSQMLQRAGLTDEKVKAYLSLHPQVLDEFVSESVSAETVEKWLKRKNNKSEDESAPKEVSRYQDTNMQGVVYELNSYIEQRLDTGGDNQLLLYELSSIIKIATKADGFALYFLGECNNSLCIFTPPGIKEGKPRLIPAGPITQGTTVSAYVAKSRKTLLVEDILGDERFPRGTGLESGTRIQSVLCLPIVTAIGDLIGILELYRHWGKEAFCLSHQEVATANLAWASVAIHQVQVCRGLAKQTELNDFLLDVSKTYFDNIVAIDSLLEHIMIYAKNLVNADRCALFQVDHKNKELYSDLFDIGEEKEGKPVFKKTKEIRFSIEKGIAGQVARTGEVLNIPDAYADPRFNREVDLYTGYTTRNILCMPIVSRGSVIGVVQMVNKISGSAFSKTDENNFKMFAVFCALALHCANMYHRIRHSECIYRVTMEKLSYHSICTSEEWQGLMQFTLPVRLCKEIELFHFDIGPFENMWPGIFVYMVHRSCGTSCFELEKLCRFIMSVKKNYRRVPYHNWKHAVTVAHCMYAILQNNHTLFTDLERKGLLIACLCHDLDHRGFSNSYLQKFDHPLAALYSTSTMEQHHFSQTVSILQLEGHNIFSTLSSSEYEQVLEIIRKAIIATDLALYFGNRKQLEEMYQTGSLNLNNQSHRDRVIGLMMTACDLCSVTKLWPVTKLTANDIYAEFWAEGDEMKKLGIQPIPMMDRDKKDEVPQGQLGFYNAVAIPCYTTLTQILPPTEPLLKACRDNLSQWEKVIRGEETATWISSPSVAQKAAASED*
